# Water-Based Pharmacophore Modeling in Kinase Inhibitor Design: A Case Study on Fyn and Lyn Protein Kinases

**DOI:** 10.1021/acs.jcim.5c01478

**Published:** 2025-09-01

**Authors:** Martin Ljubič, Marija Sollner Dolenc, Jure Borišek, Andrej Perdih

**Affiliations:** † 68913 National Institute of Chemistry, Hajdrihova 19, 1000, Ljubljana, Slovenia; ‡ Faculty of Pharmacy, 63721 University of Ljubljana, Aškerčeva cesta 7, 1000 Ljubljana, Slovenia

## Abstract

Water-based pharmacophore modeling is an emerging approach in inhibitor design that leverages the dynamics of explicit water molecules within ligand-free, water-filled binding sites to derive 3D pharmacophores for virtual screening. In this study, we assess the potential of this strategy through a case study targeting the ATP binding sites of Fyn and Lyn protein kinases—members of the Src family that have been less explored in anticancer drug discovery compared to other family members. Molecular dynamics simulations of multiple kinase structures were used to generate and validate several water-derived pharmacophores, which were subsequently employed to screen chemically diverse libraries of compounds. Two active compounds were identified in biochemical assays: a flavonoid-like molecule with low-micromolar inhibitory activity and a weaker inhibitor from the library of nature-inspired synthetic compounds. Structural analysis via molecular docking and simulations revealed that key predicted interactions, particularly with the hinge region and the ATP binding pocket, were retained in the bound states of these hits. However, interactions with more flexible regions, such as the N-terminal lobe and activation loop, were less consistently captured. These findings outline both the strengths and challenges of using water-based pharmacophores: while effective at modeling conserved core interactions, they may miss peripheral contacts governed by protein flexibility. Incorporating ligand information where available may help address this challenge. Overall, water-based pharmacophore modeling presents a promising ligand-independent strategy for identifying novel chemotypes and exploring undercharged chemical and conformational space in kinases as well as other therapeutically relevant targets.

## Graphical abstract

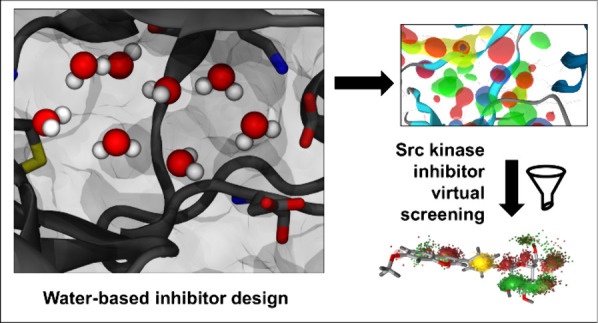

## Introduction

Protein kinases play an important regulatory role in many cellular processes such as cell apoptosis, immune regulation and development.^1^ In response to specific signals, they covalently phosphorylate and activate target proteins in the cell, thereby amplifying the signal into cellular growth cascades. These involve immune receptors such as NKG2A, whose activation mechanism has been studied previously.^1^ The family of Src protein kinases have previously been extensively studied due to their genes often being overexpressed or mutated in cancer cells, leading to their uncontrolled proliferation.^2^ Out of 32 nonreceptor tyrosine kinases known today, which act by transferring a phosphate group from the ATP molecule to the target protein tyrosine residues, 11 belong to the Src kinase family and include members such as Src, Yes, Fyn, Fgr, Lck, Hck, Blk, Lyn, Frk, Yrk, and Srms.^3^ Targeting Src kinases has been found to potentially increase vaccine efficacy,^4^ enhance immune cell cytotoxicity,^5,6^ and several drugs have been successfully developed that target these regulatory kinases and are widely used as cancer therapeutics.^7^

The structure of a typical Src kinase is composed of the myristoylation site, which enables membrane targeting and functional activation followed by a unique SH4 region. Next, the SH2 and SH3 regions serve a regulatory role and bind to a phosphorylated autoinhibition site (Figure 1A).^8^ The active kinase domain encompasses the N- and C-terminal lobes, which form the catalytic active site, which binds the ATP molecules. The C-terminal tail further contains a regulatory tyrosine residue, which stabilizes the inactive conformation by interacting with the SH2 region. The switch between the inactive to the active form involves the phosphorylation of Tyr416 in the catalytic domain, leading to changes in the orientation of the activation loop and the positioning of the αC-helix.^9^ The N-terminal lobe contains a highly conserved hinge region which binds the ATP molecule via H-bond interactions with Met345 and Glu343.^10^ The catalytically important Asp-Phe-Lys (DFG) loop faces inward in the active form and coordinates ATP and Mg^2+^ions (Figure 1B).^11^

**Figure 1:**
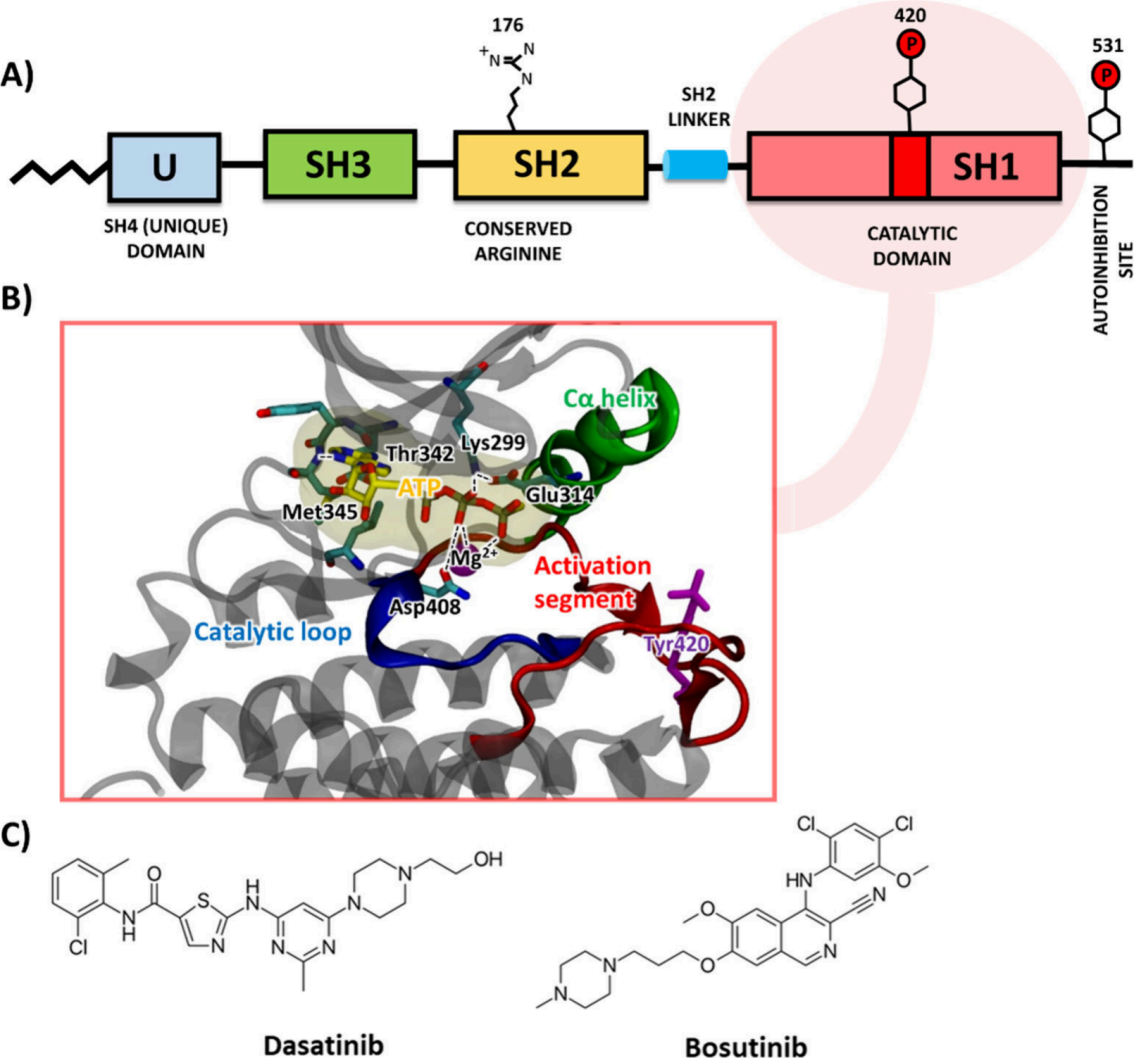
(A) Schematic representation of a structure of the Src family kinase. (B) Experimental 3D structure of the Src-protein kinase catalytic domain, which highlights key segments involved in the kinase activation and ATP binding (PDB ID: 3DQW), Fyn residue naming scheme. (C) Src-inhibitors desatinib and bosutinib used in cancer chemotherapy.

Inhibitors of Src kinases are classified into several classes. Type I inhibitors are ATP-competitive and target the active form of these kinase by predominately mimicking the adenine purine ring, but usually lack selectivity.^12^ Additionally, a deep hydrophobic pocket is adjacent to the hinge region which can be exploited to design more selective inhibitors of the active form due to the presence of a gatekeeper residue Thr342 which blocks access to the pocket in some Src-kinase active sites.^13^ Type II inhibitors bind to the inactive form and are more selective as a result, since they allow a more comprehensive exploration of this hydrophobic pocket.^10^ Several types and structural classes of Src inhibitors have been reported, and many have entered preclinical trials and some have successfully reached clinical use. Molecules such as dasatinib and bosutinib, are used in the treatment of hematologic cancers such as Acute Lymphoblastic Leukemia (ALL) and Chronic Myeloid Leukemia (CML) (Figure 1C).^7^

In the past decades, computer-aided drug discovery has been established as a successful method in drug design.^14^ With this technology, the screening of large libraries of compounds comes at a fraction of the cost required by traditional high-throughput screening (HTS) methods. The main technologies used are molecular docking and pharmacophore models. The former represent an abstract description of molecular features (e.g., spheres, planes, and vectors) that are necessary for productive molecular recognition of a ligand by a the target biological macromolecule.^15^ Since pharmacophores contain information on chemical functionalities and not atoms, virtual screening of large databases can yield chemically divergent molecules with the same effect on the investigated system enabling a more broader exploration of the chemical space.^15^

Traditionally, pharmacophore models have been generated using structures of existing ligands in ligand-based approaches or using a 3D structure of the receptor or ligand complex target.^16^ As protein structures are inherently more dynamic than a single experimental image of a complex (e.g., provided by protein crystallography), a comprehensive picture of protein–ligand interactions is often lacking.^17^ Therefore, new approaches in pharmacophore models derive and statistically analyze molecular dynamics (MD) simulations of protein–ligand complexes, which have proven to be an invaluable tool for understanding protein dynamics and solvent effects.^18^ Using this data, interaction points and pharmacophore features across an entire simulation trajectory can be extracted, providing information on the spatial distribution of these features and their occurrence frequency. Such dynamic pharmacophore models – dynophores,^19,20^ have proven valuable in optimization of hit compounds on different targets and compound classes.^21,22^

In recent years, water molecules have been exploited for the generation of pharmacophore models using simulations of solvated apo protein structures, as water can mimic key interactions a ligand might have while solvating the surface of a protein by forming hydrogen bonds and van der Waals contacts with hydrophobic patches.^23^ One approach involves the generation of dynamic molecular interaction fields (dMIFs) from the geometric and energetic properties of water molecules sampled during MD simulations. These fields can subsequently be converted into pharmacophore features using tools such as PyRod.^24^ This approach has been successfully used in the generation of pharmacophore models employed in virtual screening and can allow for unbiased mapping of interaction hotspots within the binding site, potentially revealing opportunities missed by ligand-based or static structure-based methods.^24–26^

Despite numerous classes of compounds being discovered over the last two decades using conventional and established methods, novel approaches to drug design based on pharmacophores derived from the positioning of water molecules in the binding pocket have not yet been commonly deployed in the design of inhibitors and may represent a new avenue for drug design through the exploration of apo pockets and capturing implicit solvent effects crucial for binding.^27,28^

In this study, we showcase the potential of water-based pharmacophore models in inhibitor design through a case study targeting the ATP binding sites of Fyn and Lyn protein kinases—members of the Src family that have been less explored in anticancer drug discovery compared to other family members. We conducted MD simulations on apo structures of these Src kinases to generate water-based pharmacophore models and contrasted them to the molecular recognition patterns of the two clinically used Src inhibitors. We then validated the water pharmacophore models and utilized them in a prospective virtual screening of chemically diverse libraries of compounds. The binding modes of the identified active compounds were computationally assessed in molecular simulations to determine how well they match the water-based pharmacophore predictions, thereby validating this approach as well as outlining both its strengths and limitations in rational drug design.

## Methods

### Structural Models of Src Kinases for Molecular Simulations

We constructed four models of the Src family kinase domains using experimentally determined structures available in the PDB database (Table 1): (i) Fyn (PDB: 2DQ7, active apo structure), (ii) Lyn (PDB: 2LYN, active apo structure), (iii) Src-Bosutinib (PDB: 4MXO, active form complex), and (iv) Lyn-Dasatinib (PDB: 2ZVA, active form complex). Missing loop regions in the protein structures were modeled in ChimeraX^29^ using MODELLER.^30^ For the simulations of the active hit compounds **1** and **2** bound to Lyn and Fyn crystal structures, their positions were determined by molecular docking as described later. Coordinates of all simulated systems are available in the Supporting Information.

**Table 1:** Structures of Apo and Protein–Ligand Complexes of Src Protein Kinases Lyn and Fyn Used in Molecular Dynamics (MD) Simulations

kinase	PDB ID	details
Fyn	2DQ7	active apo
Lyn	2LYN	active apo
Src + bosutinib	4MXO	active holo
Lyn + dasatinib	2ZVA	active holo
Fyn + compounds **1** and **2**	2DQ7	active apo
Lyn + compounds **1** and **2**	3A40	active holo

### Molecular Dynamics Simulations

All-atom classical molecular dynamics (MD) simulations were performed using Amber20 PMEMD package^31^ with the AMBER-ff19SB force field (FF) describing the protein atoms.^32^ The PDB 2PQR web tool was used to determine protonation states of histidine residues at Nε, Nδ, or both positions under a neutral pH condition of 7.^33^ The systems were solvated in a layer of TIP3P^34^ water molecules, extending 10 Å from the protein to the edge of the solvation box. Additionally, a small number of Na^+^ counterions were added to neutralize the systems. Topologies of the models were then prepared using the *tleap* module of Ambertools20.^31^ The final systems used in simulations were comprised of 50000–70000 atoms.

Geometries of desatinib, bosutinib and hit compounds **1** and **2** were separately optimized at the Hartree–Fock (HF) level using 6-31G­(d) basis set with Gaussian 16 program.^35^ The partial charges of the Restrained Electrostatic Potential (RESP) were generated in Antechamber (Amber20),^36^ as were the other force field parameters of the ligand, using the bond lengths and bond angles obtained from the optimized ligand geometries by applying General Amber Force Field (GAFF2). The ligand parameter files are available in the Supporting Information.

The systems were initially minimized in two steps using a steepest descent algorithm, followed by a conjugate gradient algorithm, with subsequent gradual heating to 300 K in 500000 MD steps over 300 ps with positional restraints of 100 kcal/mol Å^2^ on the heavy atoms. Next, the restraints were removed, and 10 ns isothermal–isobaric ensemble (NPT) simulations were performed, where pressure control (1 bar) was achieved using a Berendsen barostat^37^ to properly equilibrate the system before the production simulations. The production MDs were carried out in a canonical (NVT) ensemble. Temperature control (300 K) was performed using the Langevin thermostat with a collision frequency of 1 ps^–1^. The SHAKE algorithm^38^ was used to constrain hydrogen positions, and the particle mesh Ewald method^39^ with a cutoff of 10 Å was used to account for long-range electrostatic interactions. An integration time step of 2 fs was set for all MD runs.

To evaluate the dynamics and structural properties of the apo forms of Lyn and Fyn, and of these enzymes in complex with dasatinib and with bosutinib, 2000 ns long production simulations were performed for each of the models. Separately, 10 replica simulations of 10 ns or 10000 simulation frames were carried out on the Lyn and Fyn apo systems to ensure appropriate sampling at frequent intervals, which were used for the calculation of dynamic water pharmacophore models. Shorter and more numerous replicas provide better sampling of local water configurations quickly and enable more detailed exclusion volume generation, while the long simulations capture larger-scale protein motions. In molecular simulations of hit compounds **1** and **2**, docked in Lyn and Fyn the preparation of the systems fully resembled the one described above. Here, three independent replicas of 300 ns length were conducted for each prepared system.

### Analyses of Simulation Trajectories

Visual Molecular Dynamics (VMD)^40^ and PyMol^41^ software packages were used for the visualization and inspection of trajectories. MD trajectory analyses, including Root-Mean-Square Fluctuations (RMSF), Root-Mean-Square Deviation (RMSD) and calculation of cross-correlation matrices were performed with *cpptraj* module in Ambertools 20.^31^ Principal component analysis (PCA) was performed in Gromacs 19 ^42^ to extract the essential dynamics of the proteins, starting from the mass-weighted covariance matrix of the Cα and P atoms, respectively.

Dynamical pharmacophore (dynophore) models were generated with DynophoreApp on about 2000 equidistant frames of the equilibrated trajectories of the complexes between ligands and Lyn and Fyn. These calculations were performed at computers of the Molecular Design Lab at Freie Universität Berlin, Germany and subsequently analyzed and visualized in LigandScout 4.4.3.^43^

Binding free energies between Src kinases and investigated ligands were calculated using the Molecular Mechanics/Generalized Born Surface Area (MM/GBSA) method^44^ in MMPBSA.py.^45^ The value of the igb flag was set to 5, and a salt concentration of 0.1 M was used. Calculations were performed on 500 equally distant frames from the last 500 ns of the trajectories. The conformational entropic contribution of free energy was not included in the calculations, since it was previously suggested that this term does not improve the quality of the results when using MM-GBSA.^46^

### Water-Based Pharmacophore Models and Virtual Screening

The generation of pharmacophore models was conducted using PyRod version 0.7.5.^24^ Grids with a 30 Å edge lengths and a cubic shape were centered to the ATP binding sites of apo Lyn and Fyn kinases. 10000 simulation frames from the last 5 ns of each 10 ns simulation were analyzed together. Favorable regions for each pharmacophore feature placement were determined from geometric descriptors and converted into dynamic Molecular Interaction Fields (dMIFs), which we visualized in Ligandscout 4.4.3 ^43^ to observe areas of significance inside the targeted binding pocket based on water molecule occupancy. Exclusion volumes were generated for grid points with a shape score of less than 0.1. dMIFs were subsequently converted into pharmacophore features and combined with exclusion volumes into a “super pharmacophore” with the 20 highest scoring features kept of each type, which included hydrogen bond donors and acceptors, hydrophobic and aromatic interactions, and positive and negative ionizable interactions.

Next, only relevant pharmacophore features were manually selected in LigandScout and used in the generation of a combinatorial library, containing pharmacophores with between 4 and 6 features. These models were validated by Receiver Operator Characteristics (ROC) curves to identify suitable pharmacophores for the virtual screening of Lyn and Fyn protein kinase inhibitors. The library of active compounds used in validation was selected manually based on reported Src inhibitors in literature and was comprised from 100 active compounds, while the decoy set was generated using the DUD-E server (Supplementary Files). Additionally, some ligand data in the form of individual features was manually incorporated into water pharmacophore models to obtain several custom water-derived pharmacophore models.

The virtual screening of potential inhibitors was performed in LigandScout with the default settings. The compounds that matched the pharmacophore constraints were then evaluated by using the pharmacophore fit scoring function. The screening was performed on two libraries from AnalytiCon Discovery: a natural product library (MEGx) consisting of approximately 6000 purified compounds and a synthetic library (NATx) containing approximately 32000 compounds. Both libraries were previously converted with the iCon conformer to screening format with up to 25 conformations available for each compound.

### Molecular Docking Calculations

Molecular docking of hit compounds was performed in GOLD software,^47^ using experimental coordinates of the ATP binding site of active forms of Fyn and Lyn protein kinases (PDBs 2DQ7 and 2LYN). To prepare each kinase structure, hydrogen atoms were first added using the default settings. The active sites were defined with a radius of 6 Å around the ligand from the crystal structure. To generate binding poses, GOLD uses the genetic algorithm where the search efficiency was set to 200%. Among the other search settings, the following settings were applied: population size = 100, selection pressure = 1.1, number of operations = 100000, number of islands = 5, niche size = 2, migration frequency = 10. The GOLD settings used were validated by successfully reproducing the experimental dasatinib and bosutinib binding modes. The obtained docking solutions were ranked by using CHEMPLP scoring function and were visualized and analyzed in LigandScout.^43^

### Inhibition Assays of Lyn and Fyn Protein Kinases

The inhibitory effect of a selected 16 hit compounds on Lyn and Fyn kinases was assessed with KinaseProfiler (Eurofins Cerep SA, Celle-L’Evescault, France) using the radiometric kinase activity assays. Each compound was initially prepared at 50 times the final assay concentration in 100% DMSO. These working stocks were added to the assay wells as the first component of the reaction, followed by the remaining reagents, as indicated in the assay protocols.

Fyn (h) was incubated with 50 mM Tris pH 7.5, 0.1 mM EGTA, 0.1 mM Na_3_VO_4_, 250 μM KVEK­IGEG­TYGV­VYK (Cdc2 peptide), 10 mM magnesium acetate and [γ-^33^P]-ATP. The reaction was initiated by the addition of the Mg^2+^/ATP mix. After incubation for 40 min at room temperature, the reaction was stopped by the addition of phosphoric acid to a concentration of 0.5%. An aliquot of the reaction was then spotted onto a filter and washed four times for 4 min in 0.425% phosphoric acid and once in methanol prior to drying and scintillation counting.

Lyn (h) was incubated with 50 mM Tris pH 7.5, 0.1 mM EGTA, 0.1 mM Na_3_VO_4_, 0.1% β-mercaptoethanol, 0.1 mg/Ml poly­(Glu, Tyr) 4:1, 10 mM magnesium acetate and [γ-^33^P]-ATP. The reaction wa initiated by the addition of the Mg^2+^/ATP mix. After incubation for 40 min at room temperature, the reaction was stopped by the addition of phosphoric acid to a concentration of 0.5%. An aliquot of the reaction was then spotted onto a filter and washed four times for 4 min in 0.425% phosphoric acid and once in methanol prior to drying and scintillation counting.

In the standard KinaseProfiler assay, no preincubation step was included between the test compound and the kinase prior to initiating the reaction. Positive control wells contained all of the assay components except the test compound; however, 2% DMSO was included to account for any solvent-related effects. Blank wells included all reaction components with a known reference kinase inhibitor substituted for the test compound to completely abolish kinase activity and define the baseline (0% residual activity). Reference inhibitor staurosporine was used to generate the blank signal for both kinases, which was selected based on its ability to achieve complete inhibition at the concentrations used. All assays were performed in duplicate.

IC_50_ values were determined for the most promising compound through a standard 9-point dilution series with the following concentrations: 0.02, 0.063, 0.2, 0.63, 2, 6.3, 20, 63, and 200 μM. The buffer composition and activity determination followed the same procedure as the previously described inhibition assays.

## Results and Discussion

### Water-Based Pharmacophore-Guided Inhibitor Discovery: The Workflow

Water-based pharmacophore models allow the identification of key interaction features in ligand-free binding sites occupied exclusively by water molecules. By analyzing the behavior and interactions of these water molecules, such models highlight regions of favorable interactions that can guide the inhibitor design in the absence of cocrystallized ligands. This approach allows pharmacophore features to be extracted directly from the “hydration landscape”, extending the applicability of structure-based drug design to apo protein structures and uncovering interaction hotspots that may be overlooked by existing ligands or classical molecular interaction fields.

To evaluate the practical utility of the water-based pharmacophore approach, we selected Fyn and Lyn protein kinases as case studies. These Src family members play established roles in cancer-related signaling pathways^3,7^ but have been comparatively less explored as direct drug targets in anticancer drug discovery than other family members. With structural data available for both apo and ligand-bound forms, they offer a valuable starting point for assessing the potential of hydration-driven modeling.

The design of the workflow is outlined in Figure 2 with the all-atom simulations first performed starting from available apo and ligand-bound crystal structures of several kinases. The resulting trajectories were then analyzed to characterize the overall protein behavior, including flexibility and residue geometry, as well as water positioning inside the targeted binding pocket. For complexes with dasatinib and bosutinib, dynophore models were generated and binding free energies calculated to provide reference data for comparison.

**Figure 2:**
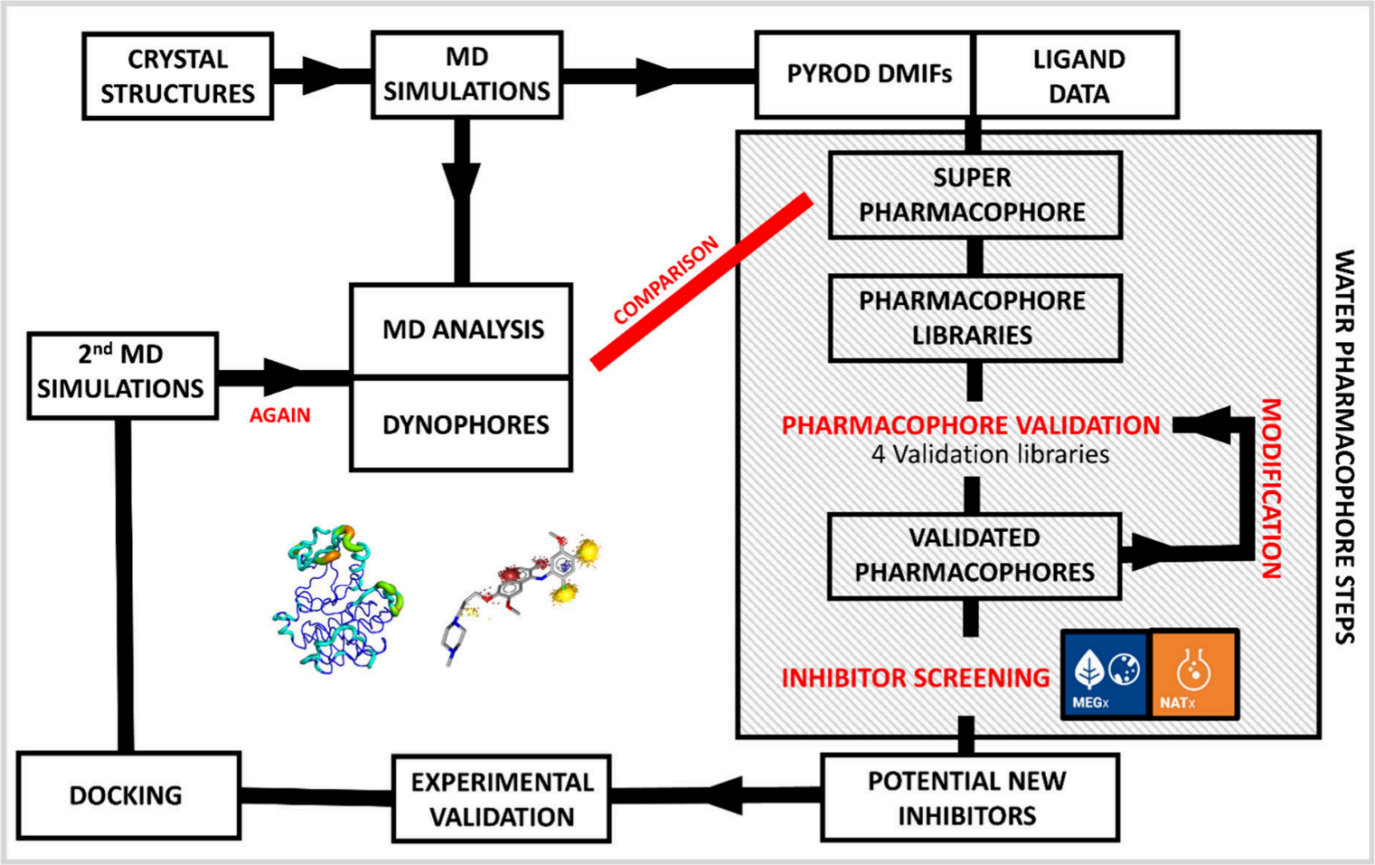
Schematic overview of the workflow for generating and evaluating water-based pharmacophore models, based on structural data of the Src kinases Lyn and Fyn. The steps in the gray box are those directly involving water-based pharmacophore models.

Water-based pharmacophore models were constructed from the dynamic Molecular Interaction Fields (dMIFs) calculated within ATP-binding pockets filled with water molecules by using the PyRod software. To reduce the complexity, a subset of only relevant pharmacophore features was manually selected from the initial superpharmacophore, and features outside the binding site were deleted. A library of pharmacophore models using a combination of initially proposed pharmacophore features was then created and screened using validation libraries, stating with a smaller handpicked selection and continuing with a larger library extracted from DUD-E and a curated library of Lck actives and decoys compiled by Guo et al.^48^ Pharmacophores with favorable selectivity and enrichment properties were used in virtual screening to identify hit compounds, which were further filtered based on the poses obtained through docking of the compounds into the ATP active sites. The most promising hit compounds were experimentally validated *in vitro* by performing radiometric kinase activity assays on both kinases.

In the final step, to provide structural context for the alignment of active hit compounds with a water-based pharmacophore, molecular docking into both kinases was followed by molecular simulations. The resulting trajectories were compared with the original dMIFs to evaluate pharmacophore-ligand overlap, dynophore patterns, and protein behavior. While primarily computational, this approach can still pinpoint the advantages as well as the current limitations of incorporating water dynamics into early stage molecular design.

### 2 Comparison of Dynamical and Structural Features Reveals a Similarity in the Src Family of Kinases and Flexibility of the ATP Active Sites

To enable comparison of target dynamics across different systems and identify trends in the behavior of selected Src family kinases, we performed 2000 ns MD simulations for both kinases using apo Fyn and apo Lyn. The structural alignment of the initial experimental protein structures revealed a high degree of similarity, particularly within the ATP binding sites (Figure 3a and S1). As a result, we expected the inhibitor design to show similar patterns in dynamics and inhibitor design.

**Figure 3:**
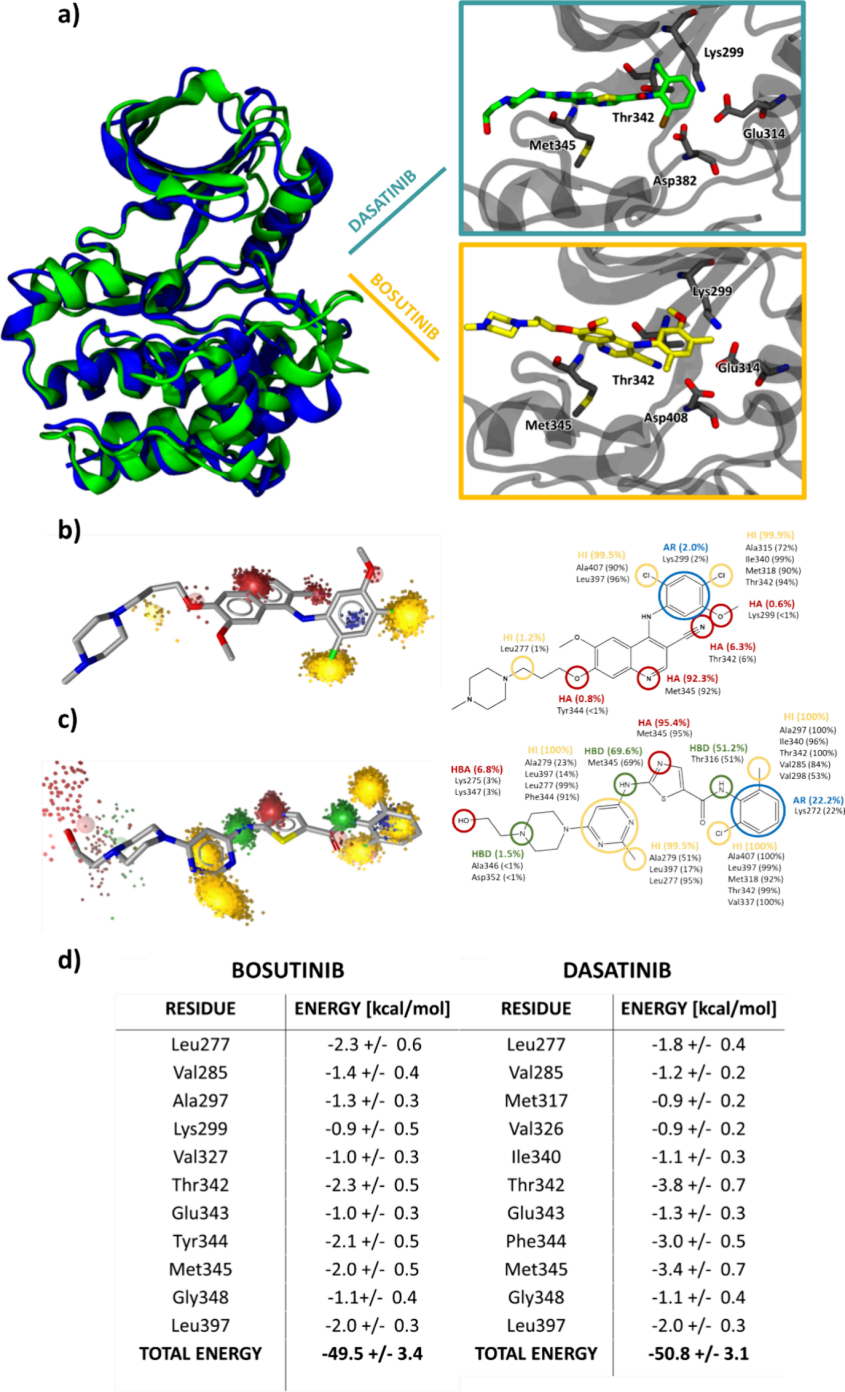
(a) Structural similarity between the main cluster of the apo active forms of Lyn (blue) and Fyn protein kinases (green). The boxes on the right show the positioning of dasatinib (lime) and bosutinib (yellow) in the ATP binding site of the active crystal structure of Src kinases. Below are dynophores of (b) bosutinib and (c) dasatinib, superimposed on the structures of these ligands. The 2D structure is shown on the right together with the total percentage occurrence during the simulation (colored) and specific contact occurrence as calculated by using Ligandscout MD analysis. HI (yellow) denotes hydrogen bonds, AR (blue) aromatic interactions, HA (red) hydrogen bond acceptors, and HD (green) hydrogen bond donors. (d) MMGBSA per-residue contributions of the top 11 residues in Src-bosutinib and Lyn-dasatinib.

RMSD values were comparable between the two apo structures with the average values at 2.5 Å (Figure S2). Principal component analysis (PCA) scatterplots indicated that sampling of the trajectories was sufficient, with the top two principal components accounting for 20–40% of the total variance in most simulated models (Figures S3 and S4). The Lyn-dasatinib PCA scatterplot was somewhat irregular, indicating suboptimal sampling, although visual inspection did not show any abnormalities compared to every other model. Correlation graphs also displayed the same patterns across all structures (Figure S5), with the protein structures containing ligands displaying slightly lower correlations and anticorrelations. The overall global dynamics of the apo models were consistent. The first ten residues of each system did display a much higher flexibility at the top of the N lobe; however, it should not have a significant impact on the binding site. These calculations suggest that the two kinases display the same general behavior, particularly at the ATP binding site.

However, equilibrated structures during the simulations adopted a slightly different conformation of the Lys299 residue compared to the crystal structures (Figure S6). This residue in the active form interacts with Glu314, and was positioned lower in the simulations. This conformational shift likely affects the positioning of water molecules and thus the generation of pharmacophore models, showcasing a degree of plasticity in this region that adapts to the ligand structure when adopting an appropriate conformation.

To complement the apo-site analysis, we further performed 2000 ns MD simulations of Src in complex with bosutinib and Lyn in complex with dasatinib. The former was selected because of its ability to incorporate water molecules in its binding, and the latter due to being a potent Src inhibitor found in many clinical trials. The c-Src complex with bosutinib was selected because of the lack of Fyn or Lyn structures with this ligand. Due to the structural similarity and highly conserved sequences of Src family kinases, we could expect to observe the same binding mode compared to Lyn and Fyn (Figure S7). The protein backbone RMSD stabilized below 2.5 Å in both systems, while the ligands remained stably bound in the ATP binding pocket, with average ligand RMSD values around 1.1 Å (Figure S8). The Lyn–dasatinib complex exhibited slightly greater overall stability, with lower fluctuations throughout the trajectory.

To evaluate the influence of ligand binding on local kinase dynamics, we analyzed the fluctuations at the residue level using RMSF plots (Figures S9 and S10). On average, ligand-bound systems showed a slightly reduced flexibility compared to their apo counterparts, especially in the activation loop and the DFG motif. This local stabilization is likely a direct consequence of ligand binding. Importantly, these dynamic differences may translate into different water molecule patterns within the binding site, leading to different pharmacophore features. While the presence of a ligand may restrict hydration to conformations consistent with its own shape, simulations of apo structures may reveal alternative interaction hotspots not captured by existing inhibitors.

Additionally, we evaluated the total binding free energy of the ligand-bound structures (Figure 3d). Experimentally, both molecules exhibit strong binding to Src kinases, with a determined IC_50_ value of 8.5 nM for dasatinib to Lyn and sub 1.5 nM for bosutinib to c-Src, with similar values for Fyn and Lyn.^49,50^ The binding free energy of bosutinib was −49.5 ± 3.4 kcal/mol and that of dasatinib −50.8 ± 3.1 kcal/mol. Significant contributions to the binding energy of dasatinib come from the hinge methionine (−3.4 kcal/mol) and gatekeeper threonine (−3.8 kcal/mol) residues, while the per-residue contributions to bosutinib binding were lower, despite only having a marginally smaller binding free energy. Bosutinib has a relatively unique aspect of binding as it creates a water bridge with Glu314, thus achieving energetically favorable binding despite having fewer hydrogen bonds with the hinge region than some other inhibitors (Figure S11).^51^ This proves that efficient water networks can enhance the ligand binding efficiency.

Overall, the initial set of simulations showed that Fyn and Lyn members of the Src family kinases exhibit a high degree of similarity. While the global structures remained conserved, subtle differences in flexibility and residue positioning were observed between the inhibitor-bound and apo structures, hinting at the possibility of finding new alternative ligand binding modes with water pharmacophore models.

### Water-Based Pharmacophores and Dynophores Offer Insight into Possible Inhibitor Interaction Patterns

We generated water-based pharmacophore models using water dynamics in the ATP binding site of apo Lyn and Fyn based on performing 10 replicas of 10 ns long simulations per kinase. we observed that water pharmacophore H-bond and hydrophobic interaction dMIFs were able to successfully predict interactions with the hinge region of the ATP binding site where the adenine portion of ATP typically forms H-bonds with the protein, particularly Met345 (Figure 4A).

**Figure 4:**
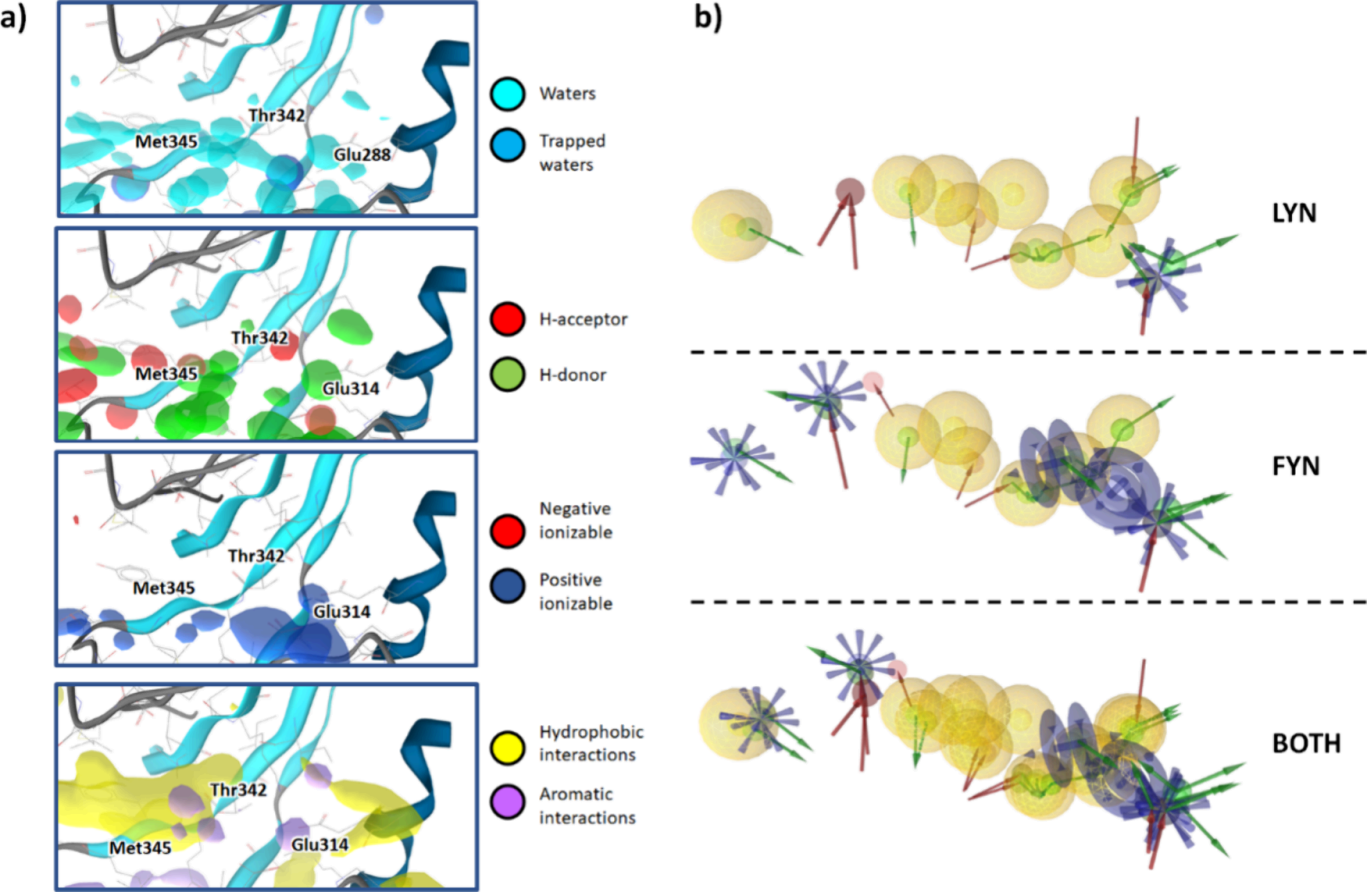
(a) Showcase of dynamic molecular interaction fields (dMIFs) determined on apo Fyn kinase: waters with at least 50% occurrence (cyan), trapped waters with 20% occurrence (blue), hydrogen bond acceptors (red), and hydrogen bond donors (green) with at least 40% occurrence, positive (blue) and negative (red) ionizable interactions with at least 40% occurrence, and hydrophobic (yellow) and aromatic (purple) interactions with at least 40% occurrence. (b) The most relevant features of pharmacophore models of Lyn and Fyn, as well as all pharmacophore models aligned for better comparison, converted from the dMIFs.

In many Src inhibitors, bonds with NH or carbonyl groups in the Met345 main chain and the Glu343 carbonyl oxygen are common.^52^ Critical in kinase inhibitor design is the gatekeeper residue Thr342 residue. In Src kinases, this short residue does not block access to the hydrophobic pocket, which can give rise to more specific inhibitor design, alongside acting as a hydrogen bond acceptor, as confirmed with water pharmacophore models. Water pharmacophores also predicted potential H-bonds to Ala394, Asn395, Asp408, Ile340 and Asp352. The dMIF representing hydrophobic interactions was located near the hinge region but did not extend far into the hydrophobic pocket. This is likely because of the positioning of Lys299, which resides slightly lower in the simulation trajectories than in the crystal structure (Figure S6).

The initial Lyn and Fyn super pharmacophore models, generated from the dMIFs, were then compared to each other (Figure 4b). We expected the pharmacophore models to exhibit the same characteristics due to the conserved ATP binding site. The hydrophobic pharmacophore features were nearly identical upon comparison of Fyn and Lyn. The hinge region and adjacent area of the hydrogen bonding network were also conserved, confirming our predictions. Some differences were still observed at the extremities of the binding site, where protein sequences are slightly less conserved. Knowing the similarities, we narrowed our focus purely on super pharmacophore models derived from Fyn with the assumption based on the observed behavior in our simulations that screened compounds would likely display similar binding to all Src kinase ATP binding sites.

One important limitation we observed was that when attempting to generate pharmacophore models from larger trajectories, they were somewhat suboptimal. While generated pharmacophore features are comparable to using shorter trajectories, exclusion volumes are far scarcer and sometimes failed to generate entirely, unless the threshold for exclusion volume generation is set to a higher number. In the 2000 ns trajectory initially intended for water-based pharmacophore generation, exclusion volumes were not created under a cutoff value of 20, while an expected pattern was observed when shorter trajectories with many replicas were used and a cutoff of 0.1. This suggests that the increased mobility and deviations from the initial structure may represent a challenge in the alignment of frames in the protein trajectory, thus making exclusion volume sphere generation challenging in very long trajectories with spaced-out frames.^53^ Alternatively, it may also suggest that mobile protein regions may present challenges for water pharmacophore usage as regions that are less accessible to water molecules in some trajectory frames may fail to produce adequate dMIFs.

For further insight, we took advantage of the dynophores calculated for the ligand-bound structures, which revealed that bosutinib formed persistent hydrophobic interactions and one important H-bond acceptor interaction (Figure 3b,c). Interestingly, the acceptor that formed the important water bridge to Glu314 formed an H-bond in only 6.3% of the trajectory. This feature has been found to play a critical role in bosutinib binding.^51^ Both, dasatinib and bosutinib, formed strong hydrophobic interactions which were present throughout the simulations and a strong H-bond acceptor, with dasatinib forming overall more interactions with the hinge region of the binding pocket. Water molecule analysis of the bosutinib simulation showed that waters were not trapped in this bond but broke and reformed it at a relatively fast rate. We noticed the presence of one or two water molecules in this region at a time and even a third molecule could be observed in some frames, additionally strengthening the hydrogen bonding network between bosutinib and the active site ().

While PyRod is not able to explicitly detect water networks, trapped water dMIF indicated the possibility of trapped waters around this area (Figure 4a). This aligns with a strong correlation between occupancy rates of water molecules in apo structures and experimentally observed waters in protein–ligand structures.^54^ This suggests that dynamic molecular interaction fields can be another useful tool to detect important water molecules that can be exploited during inhibitor design. The importance of trapped waters in bosutinib complex is known^51^ and has now been alluded to with the use of water pharmacophores and in the future, more systems may be thoroughly explored by tracing water molecules, allowing for more comprehensive binding site exploration.

### From Water-Based Pharmacophores to Experimental Hits: Validation and Screening of Lyn and Fyn Kinase Inhibitors

From the super pharmacophores, we generated pharmacophore libraries and screened them against validation libraries of active and decoy molecules. Preliminary screening was performed on a smaller library of handpicked Src inhibitors collected from the literature consisting of 89 compounds and 120 decoys generated with the DUD-E decoy Web server. The secondary handpicked validation set consisted of the same active compounds and all 6000 decoys created with DUD-E to further narrow down the pharmacophore selection. The final selection was done using by the Lck validation set created by Guo et al.^48^ with 1000 curated active compounds and 3000 decoy compounds and the ChEMBL validation set from DUD-E with 920 actives and 27000 decoys.^55^ Lck was selected due to an abundance of ligand information and structural similarity to Fyn and Lyn and inhibitors were presumed to have a similar inhibitory pattern across all Src kinases.

In the end, pharmacophores **A**, **B**, **C**, and **D** were selected based on their validation statistics (Figure 5) as well as their prospective properties for virtual screening of Src inhibitors, while containing different selections of features. Details regarding pharmacophore creation, validation statistics, and screened hits are more extensively described in the Supporting Information (Figure S12).

**Figure 5:**
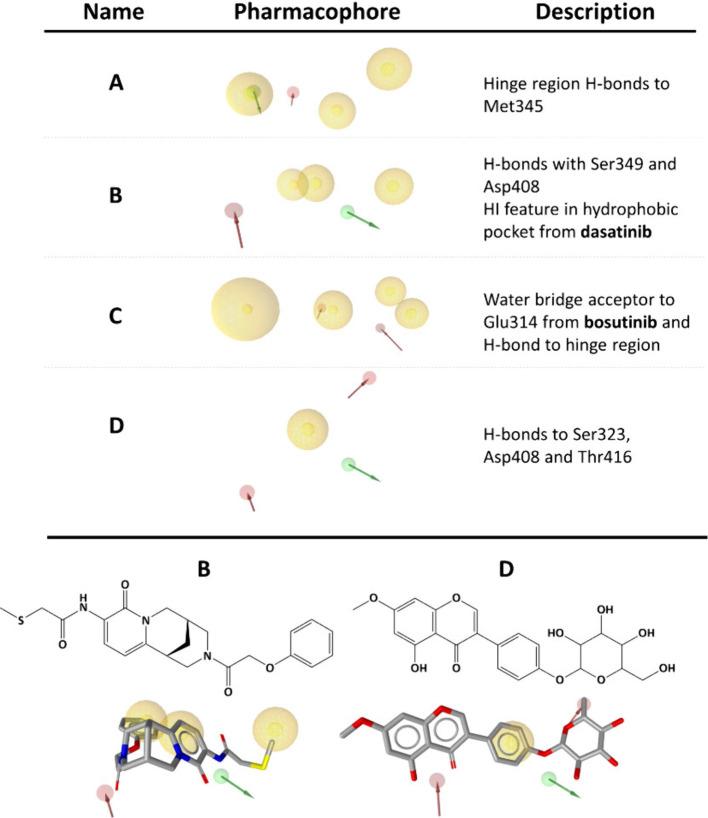
Selected pharmacophores **A**–**D** are used for virtual screening of potential Src kinase inhibitors. Pharmacophores **A** to **D** represent screening pharmacophores derived from the Src kinase active structures. Examples of two relevant compounds obtained from pharmacophores **B** and **D** is shown. Additional validation statistics are included in the Supporting Information (Figure S12).

Pharmacophore **A** was the result of a two-step process: filtering pharmacophores in step 1 based on its hydrogen bonds and adding hydrophobic interactions for secondary validation using the same validation libraries. The resulting pharmacophore performed excellently when screened with the handpicked library (EF = 139.5) and moderately with the other two larger libraries.

Pharmacophore **B** was a custom pharmacophore that included a hydrophobic interaction feature from the dasatinib structure. This feature was added to compensate for the lack of hydrophobic features inside of the hydrophobic pocket past the gatekeeper residue, which can provide selectivity over other kinase types with more obstructive gatekeeper residues. Additionally, the hydrogen bond features are formed to Asp408 and Ser349 and are not to the typical hinge region residues such as Met345 or gatekeeper The342 which ligands such as dasatinib and bosutinib utilize.^52^ Validation with the handpicked library was promising (EF = 30.8) but less so with the larger and less curated libraries.

Pharmacophore **C** featured the bosutinib H-bond acceptor, which is used by the ligand to form a water bridge consisting of two water molecules as well as a standard hinge region H-bond acceptor feature. Among the hydrophobic interactions, a large sphere was manually added to ensure that the virtual hits are properly elongated. This pharmacophore exhibited better validation statistics with the larger ChEMBL set (EF = 6.4) than with the handpicked set, potentially displaying a less specific profile for general hits but lower selectivity for established inhibitor types, which was reflected in the larger number of screened hits.

Pharmacophore **D** featured a modified pharmacophore B, with hydrogen bonds to Asp408 and Ser349 and an additional H-acceptor above the Thr342 gatekeeper with potential donor interactions to neighboring residues. The larger focus on hydrogen bonds in this pharmacophore allows us to probe more specific molecules, but the consequence is having worse validation statistics (ChEMBL set EF = 2.0).

Pharmacophore models **A**–**D** were used in virtual screening employing two compound libraries: one composed of natural products and another of synthetic compounds, which were developed using features of natural products and their moieties. This approach was intended to explore a wide region of chemical space enriched in the unique molecular characteristic of natural products, which often contribute to favorable bioactivity and structural diversity. Overall, about 35000 compounds were used in screening. In the end, the total number of 83 screened hits from both libraries were identified, which were narrowed down to a final selection of 16 diverse compounds. These compounds were selected to represent a diverse set of scaffolds, enabled by the fact that water pharmacophores did not require specific ligand information in the initial modeling step (Table S1).

A final set of diverse 16 hit compounds **1**–**16** was screened *in vitro* in Fyn and Lyn inhibition assays to evaluate inhibitory effects as described in the Methods section (Tables 2 and S1). Compound **1**, a flavonoid-based compound from the library of natural products, and compound **2** from the synthetic library of nature inspired compounds with a less frequently used 1,2,3,4,5,6-hexahydro-8*H*-1,5-methanopyrido­[1,2-*a*]­[1,5]­diazocin-8-one core scaffold were identified as potential inhibitors. Compound **1** has also previously been identified as Prunetrin, and has been shown to have potent anticancer effects on HepG2 and Huh7 cells.^56^ The residual activity of Fyn and Lyn at an inhibitor concentration of 10 μM was 42% and 5%, respectively, for compound **1**, marking the compound as a promising Src inhibitor. Compound **2** at the same concentration resulted in 50% and 74% residual activities, suggesting a weaker inhibitory effect. For compound **1**, we further determined the IC_50_ value, which was 8.7 μM for Fyn and 4.4 μM for Lyn, implying convincing inhibitory activity in the low micromolar range. Overall, the hit rate of our screening campaign was 12.5%, which is comparable to the success rates generally expected for prospective virtual screenings.^57^ These results confirm the ability of water-based pharmacophore models to prioritize meaningful candidates from chemically diverse libraries, thus showing promise in uncovering new classes of compounds that can bind to the target. However, a more comprehensive *in vitro* campaign would have to be conducted to fully understand its applicability.

**Table 2:**
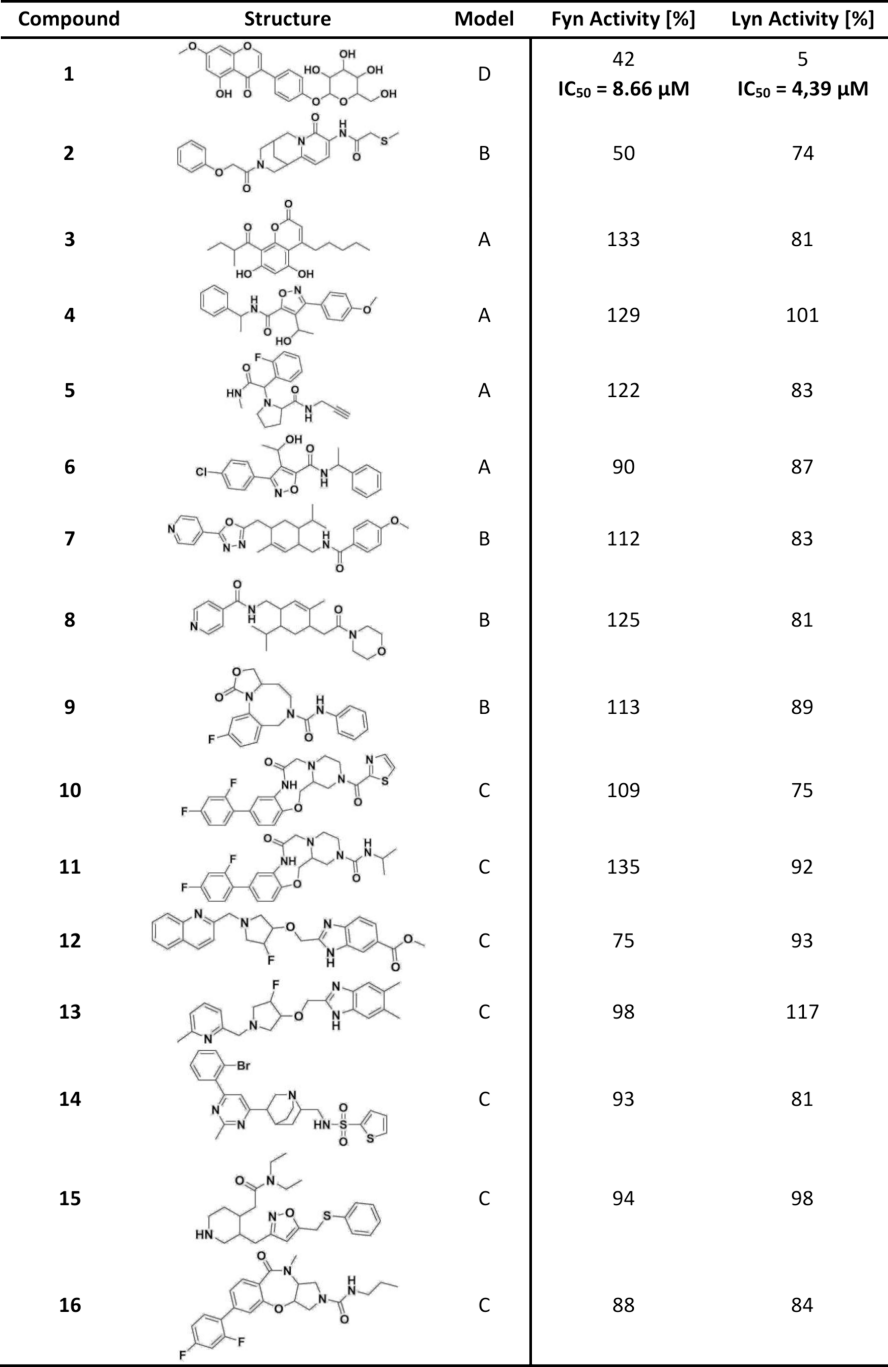
Compounds Selected for *In Vitro* Testing as Prospective Fyn and Lyn Kinase Inhibitors with the Pharmacophore Model from Which They Were Derived^a^

a Activity values from inhibition assays are displayed on the right. Additionally, we determined the IC_50_ values for the most promising compound.

### Computational Analysis Reveals Strengths and Limitations of Water-Derived Pharmacophores in Capturing Ligand Binding Mode

Beyond evaluating the utility of the water-based pharmacophore approach to identify novel kinase inhibitors, we were also interested in correlating the orientations and interactions of active compounds from virtual screening with their binding in the ATP binding site of both kinases. By performing this comparison, we aimed to determine whether key interaction features are preserved in the predicted binding poses of the screened hits.

First, we docked active compounds **1** and **2** into the ATP binding sites of apo Lyn and Fyn. A predominant binding pose was observed for the stronger inhibitor flavonoid **1** with the hydroxyl-rich monosaccharide moiety reaching deep inside the ATP binding pocket, forming several potential hydrogen bonds with the activation loop (Ala407, Phe409), αC-helix (Glu314) and the gatekeeper residue Thr342. On the other hand, docking of compound **2** yielded several distinct poses, reflecting its higher degree of conformational flexibility. Here, we selected the most frequent pose possessing a distinct V-shape orientation of the methanopyrido­[1,2-*a*]­[1,5]­diazocin-8-one core heterocycle in the binding pocket (Figure 6). These two poses were selected as starting points for molecular simulations, which were for each protein kinase performed in three independent replicas, each in the length of 300 ns. This allowed for better sampling of the available conformational landscape in the evaluation of inhibitor binding.

**Figure 6:**
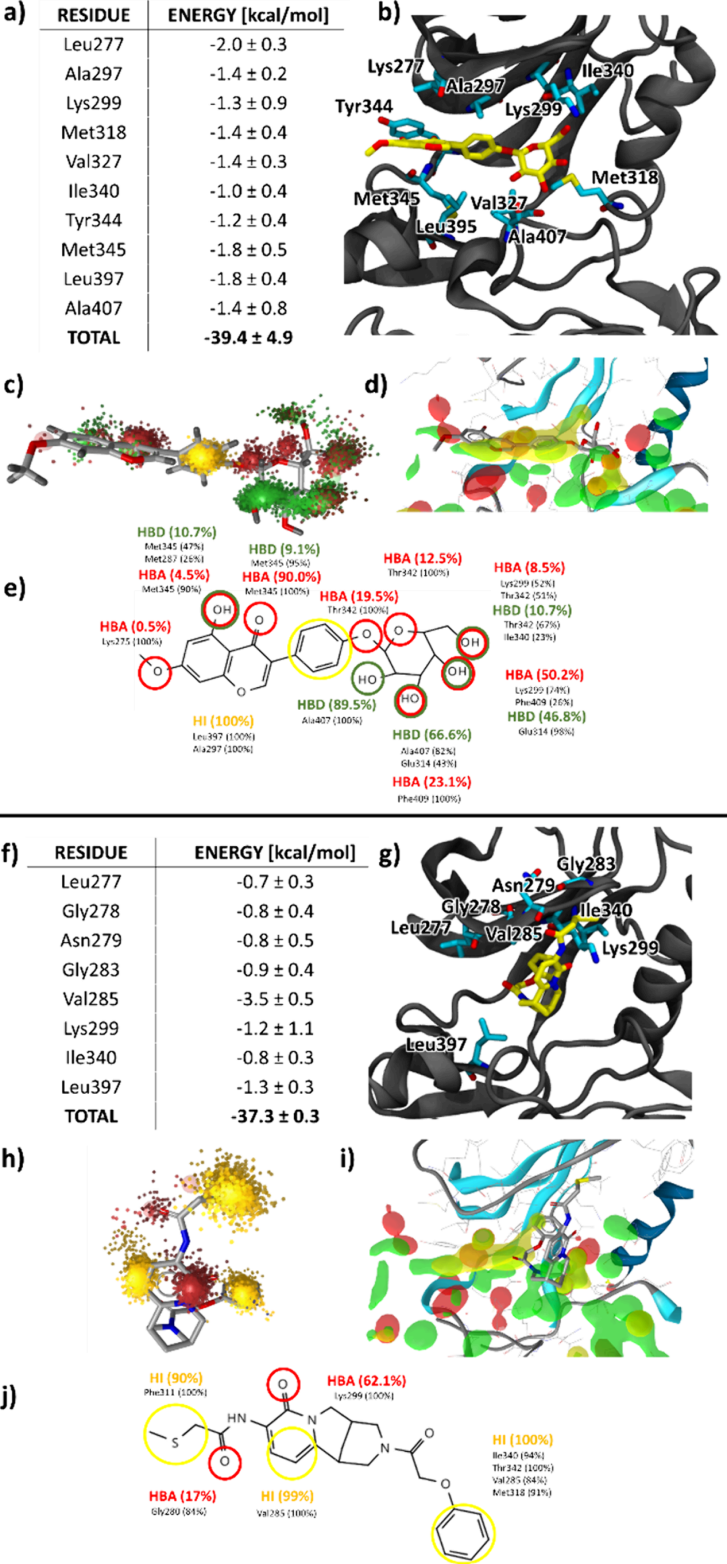
Computational validation of the binding properties of active compounds **1** (top) and **2** (bottom) in the binding pocket of Fyn kinase using the replicas with the most favorable binding. (a) MM/GBSA per-residue decomposition of the most significant Fyn residue contributions to the binding energy. (b) Binding pose of compound **1** in the Fyn kinase ATP binding site. The important residues are shown in a licorice representation. (c) Dynophore model of compound **1**. (d) A comparison of compound **1** position inside the binding pocket relative to the dMIFs which were used to construct water-based pharmacophore models. (e) 2D representation of the compound **1** dynophore model with the interaction pattern constructed from the percentage occurrence of each pharmacophore feature. (f) MM/GBSA per-residue decomposition of the most significant Fyn residue contributions to the binding energy. (g) Binding pose of compound 2 in the Fyn kinase ATP binding site. The important residues are shown in a licorice representation. (h) Dynophore model of compound 2, calculated using the MD trajectory. (i) A comparison of compound 2 position inside the binding pocket relative to the dMIFs which were used to construct water pharmacophore models. (j) 2D representation of the compound 2 dynophore model with the interaction pattern constructed from the percentage occurrence of each pharmacophore feature.

Visual inspection of trajectories revealed the same general pose for compound **1** in both Fyn and Lyn, with moderate differences in the positioning inside the binding pockets, with the ligand pointed toward the αC-helix (Figure S13). RMSD values indicated that the flavonoid was relatively unstable in the first half of the trajectory but settled in stable conformations afterward and maintained stability throughout the rest of the simulation (Figure S14). In one replica, the ligand left the binding pocked but soon reentered the pocket in a slightly different configuration. Energetically, Fyn and Lyn complexes with bound flavonoid **1** yielded similar values with an average binding free energy of −36.5 and −39.4 kcal/mol across all replicas (Table S2). This was roughly 10 kcal/mol less favorable than bosutinib and dasatinib binding, which can explain the lower IC_50_ values determined for the two drug molecules. While active compound **2** also remained bound throughout the simulations, it adopted several distinct configurations, showing much larger flexibility. Furthermore, binding free energies were notably lower than those for the natural product compound **1** (−29.7 kcal/mol for Fyn and −30.8 kcal/mol for Lyn), which is consistent with its weaker inhibition observed for both studied kinases.

From the replica simulations for both compounds, we selected the system with the most favorable binding energy in the second half of each Fyn trajectory and calculated dynophore models, which showcase the compounds’ interaction patterns across the simulation trajectories, and compared them to the original dMIFs which were used to create water-based pharmacophore models for virtual screening (Figure 6). We can observe that although after the simulations compound **1** did not fit as well to screening pharmacophore model **D**, most of the interactions with the ATP binding pocket can still be explained by the dMIFs that were later converted into pharmacophore features. A large part of the ligand’s aromatic rings fit well into the hydrophobic region predicted by PyRod. H-bonds with the hinge region such as Met345 were also predicted as well as H-bonds with the αC-helix and activation loop which the ligand formed on its polar side with its OH groups (Figure 6d). In the same way, we computed dynophore models for the lowest energy replica simulations of Lyn and found that the interaction patterns of compounds **1** and **2** followed the same general trends as those seen in the Fyn simulations. This agreement can be attributed to the similarities in protein structures and comparable positioning of the ligands in the binding pockets during the simulations (Figure S15).

One area in which the water pharmacophores were particularly less successful at predicting features was the flexible loop and adjacent section of the N-terminal lobe outside the hinge region present in both kinases (Figure 1b) for which our binding models showed that it was capable of accommodating and favorably interacting with both active ligands. This was apparent particularly in the case of compound **2**, which formed most of its interactions with this region as identified through the generated dynophore models (Figure 6g). This includes residues such as Val285, which had the largest contribution to the binding free energy of inhibitor **2** through hydrophobic interactions but was not identified through dMIFs or Ile340, which formed interactions with both ligands and was absent from the water pharmacophore models. We see that the binding mode of compound **2** was very distinct and did not include interactions with the hinge region or the activation loop. It also included fewer H-bonds with the ligand, the only prominent one being a hydrogen acceptor from Lys299, a key residue involved in kinase activation. From the binding pose, we can observe that the interactions with the binding site were therefore not fully utilized. This was even more apparent in other replicas, which had much lower binding energies where the ligand formed a folded structure, which formed fewer interactions with the protein.

Molecular simulations thus revealed that compound **2** was capable of forming hydrophobic interactions that could not be predicted from the dynamics of water molecules. It is possible that in the regions with higher plasticity, the protein can adapt its structure to better accommodate the ligand, but these regions cannot be accessed by water molecules alone, showcasing a limitation in using this approach. It is possible that these regions were not predicted because of the use of shorter simulations to allow for exclusion volume generation, sacrificing some potential features that could have been spotted in longer simulations. Furthermore, proteins can exhibit different conformations in apo and ligand-bonded forms and in the absence of a ligand, and some of these configurations are possibly too uncommon to detect in such short and unbiased simulations as interaction between pairs of molecules involve mutual structural rearrangements guided by a convoluted energy landscape.^58^ Kinases in particular display large conformational plasticity through their reversible mechanism of activation and inactivation, presenting a challenge in inhibitor design.^59^

Building on these observations, we noticed that upgrading the water-based pharmacophores with available ligand- or structure-based information leads to more comprehensive and predictive screening models. Indeed, by examining known ligand-bound complexes of bosutinib and dasatinib, we gained a deeper understanding of the binding site and were able to incorporate this information into refined pharmacophore models. Our findings further demonstrated that exploiting water molecules for drug design can help us discover promising new classes of compounds, as it does not require prior information and can be used on apo structures of proteins as well as being able to uncover potential new interactions purely based on water interactions with the empty binding pocket. Hydration of the binding site plays a significant role in the activation mechanism of kinases, further highlighting the potential of using water molecules to uncover inhibitors.^60^ We have nevertheless also investigated its ability to predict the same patterns as observed in the known ligand-bounded structures of bosutinib and dasatinib and successfully validated its ability to predict most of the important interactions with the hinge and ATP-site proximal regions through dMIFs. A major challenge was converting them to usable pharmacophore models, particularly hydrophobic and aromatic dMIFs, which may generate a large number of features that result in large numbers of plausible pharmacophore models. Finding optimal models from these can present a challenge, especially when trying to discover less common binding modes. Additionally, a large conformational flexibility of certain protein regions, which can structurally adapt to ligands, can present a challenge, as these conformations are rare in apo form simulations.

## Conclusion

Harnessing the dynamic behavior of water molecules in protein binding sites represents a valuable new addition to the medicinal chemist’s toolkit of rational molecular design. In this study, we demonstrated the utility of water-based pharmacophore models derived from dynamic molecular interaction fields (dMIFs) in identifying novel inhibitors of the Src family kinases Fyn and Lyn, two relatively understudied targets in the context of anticancer drug discovery. Through virtual screening of validated water-based pharmacophores, we identified diverse chemical classes of hit compounds, of which a flavonoid-based compound **1** from the purified natural compounds screening compounds library exhibited low-micromolar inhibitory activity on both kinases while a nature-inspired synthetic compound **2** with the methanopyrido­[1,2-*a*]­[1,5]­diazocin-8-one core showed weak inhibition but can be considered a potential starting point for further optimization. These results confirmed that water-based pharmacophore modeling, as implemented in PyRod, can effectively gain new insight into inhibitor design and uncover new classes of compounds.

The effectiveness of directly exploiting water molecules for inhibitor design of selected kinases was evaluated by comparing data from molecular simulations of two clinically used Src inhibitors and additionally performing simulations of both active compounds to the Fyn and Lyn ATP active sites. Computational analysis revealed that key predicted interactions, particularly with the hinge region and ATP binding pocket, were retained in the bound states of these hits. However, interactions with more flexible regions such as the N-terminal lobe and activation loop were less consistently captured. Water-based pharmacophores, while effective at modeling of the conserved core interactions, may thus miss peripheral contacts governed by protein flexibility, and incorporating ligand information where available may help address this challenge. Our study highlights how leveraging water dynamics can enrich the landscape of possible pharmacophore models and presents a promising ligand-independent strategy for identifying novel chemotypes and exploring uncharted chemical and conformational space in kinases as well as other therapeutically relevant targets.

## Supplementary Material





## Data Availability

Molecular simulations, analysis and visualization were performed with Amber20, AmberTools20, VMD 1.9.3, PyMol 2.0, PyRod 0.7.5, and ChimeraX. Molecular docking was performed with GOLD and pharmacophore modeling with Pyrrod and Ligandscout. All procedures and workflows are described in the Methods section. Structure and parameter files are provided in the Supporting Information. Additional data, including input files, final structures, ligand parameter files, and trajectories, are available at Zenodo: 10.5281/zenodo.15483954

## Notes

Structural comparison of the aligned structures of Fyn and Lyn, RMSD and RMSF values, B-factor flexibility representations, PCA scatterplots, cumulative contributions of PCs and correlation analysis of the simulated systems, comparison between the crystal and simulation structures of Fyn, multiple sequence alignments, representative snapshots of the Src-bosutinib binding site, pharmacophore model details, additional dynophore models, representative structures of compounds in all replicas, supporting table of the library of small organic molecules from the NATx and MEGx libraries, analytical data for active hit compounds (PDF)
Coordinates of all initial structures for MD simulations including AMBER ligand parameter and topology files (ZIP)
