# Supplementary material for: Water-Based Pharmacophore Modeling in Kinase Inhibitor Design: A Case Study on Fyn and Lyn Protein Kinases
Source: J Chem Inf Model. 2025 Sep 1;65(18):9747–61. doi: 10.1021/acs.jcim.5c01478 (PMC12458683; doi:10.1021/acs.jcim.5c01478)
Supplement: Supplementary file 1 [file ci5c01478_si_001.pdf]

SUPPORTING INFORMATION

Water-Based Pharmacophore Modeling in Kinase Inhibitor Design: A Case Study on Fyn and Lyn Protein Kinases

Martin Ljubič[†,&],Marija Sollner Dolenc[&] , Jure Borišek[†,*], Andrej Perdih[†,&,*]

[†]National Institute of Chemistry, Hajdrihova 19, 1000, Ljubljana, Slovenia

[&]Faculty of Pharmacy, University of Ljubljana, Aškerčeva 7, 1000 Ljubljana Slovenia

Corresponding author*:

E-mail: jure.borisek@ki.si

E-mail: andrej.perdih@ki.si

Table of contents

Figure S1.	3
Figure S2.	4
Figure S3.	5
Figure S4.	6
Figure S5.	7
Figure S6.	8
Figure S7.	9
Figure S8.	10
Figure S9.	11
Figure S10.	12
Figure S11.	13
Figure S12.	14
Figure S13.	15
Figure S14.	16
Figure S15.	17
Table S1.	18
Table S2.	19
Analytical data for active hit compounds.	20

Supporting figures

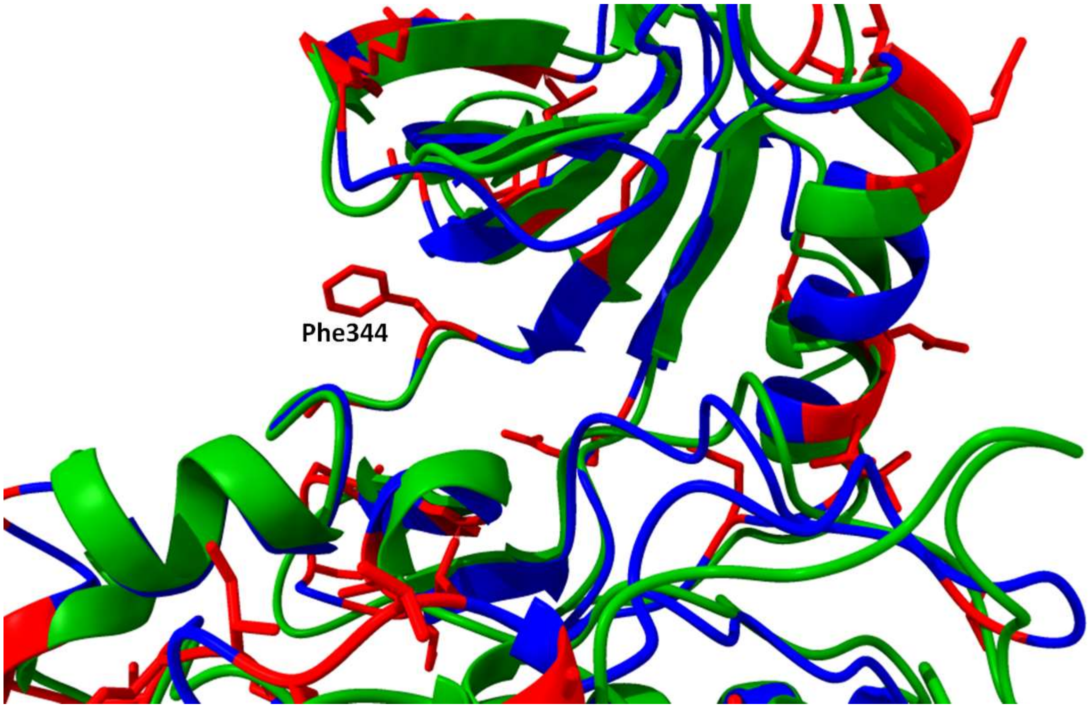

Figure S1. Structural comparison of the aligned structures of Fyn (green) and Lyn (blue). The Lyn residues that differ from Fyn are painted red. A strong conservation of the ATP binding site can be seen from the image, the only significant differing residue being a substitution of Phe344 in Lyn from Tyr in Fyn outside the main ATP binding pocket.

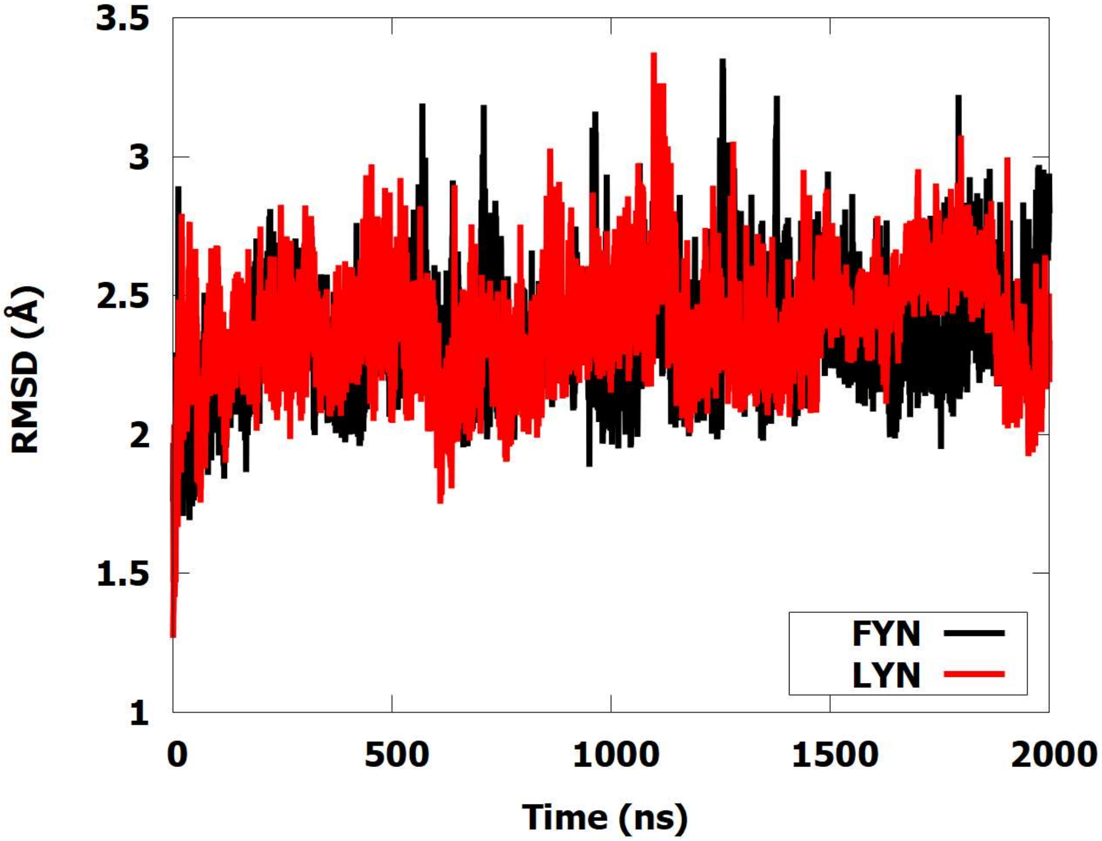

Figure S2. RMSD values for Lyn (red) and Fyn (black) kinase during 2000 ns long MD simulations runs.

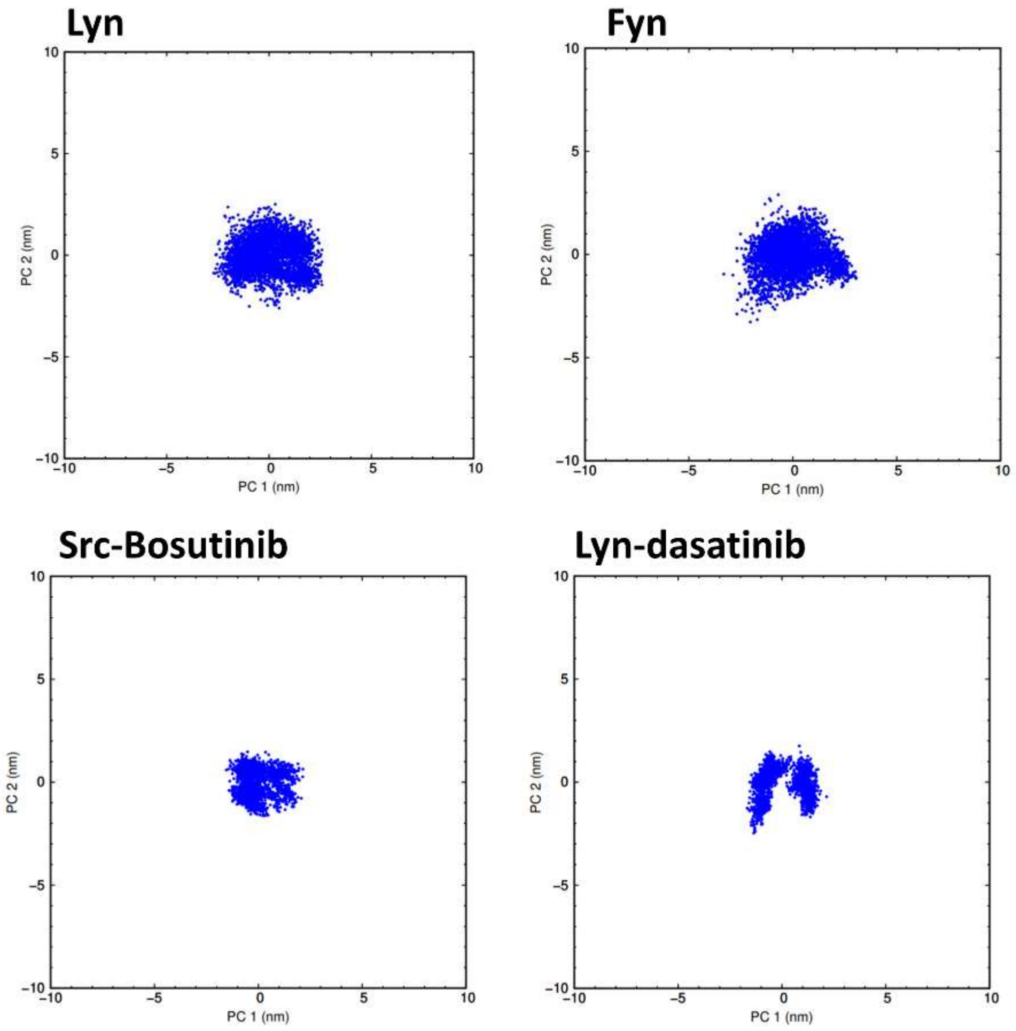

Figure S3. PCA scatterplots of all four protein systems (Lyn, Fyn, Src-Bosutinib and Lyn-dasatinib) from the simulated trajectories of 2000 ns long MD simulations runs.

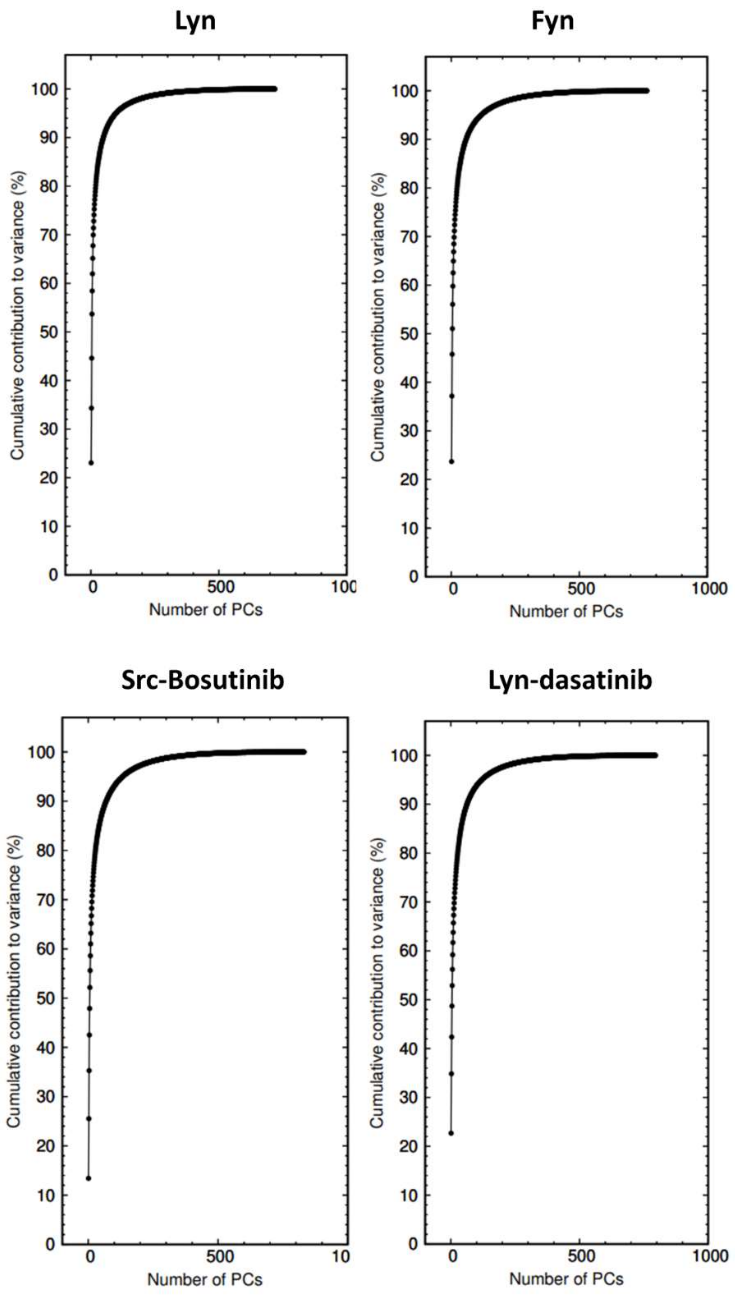

Figure S4. Cumulative contributions to the total variance by each of the PC's in Lyn, Fyn, Src-Bosutinib and Lyn-dasatinib.

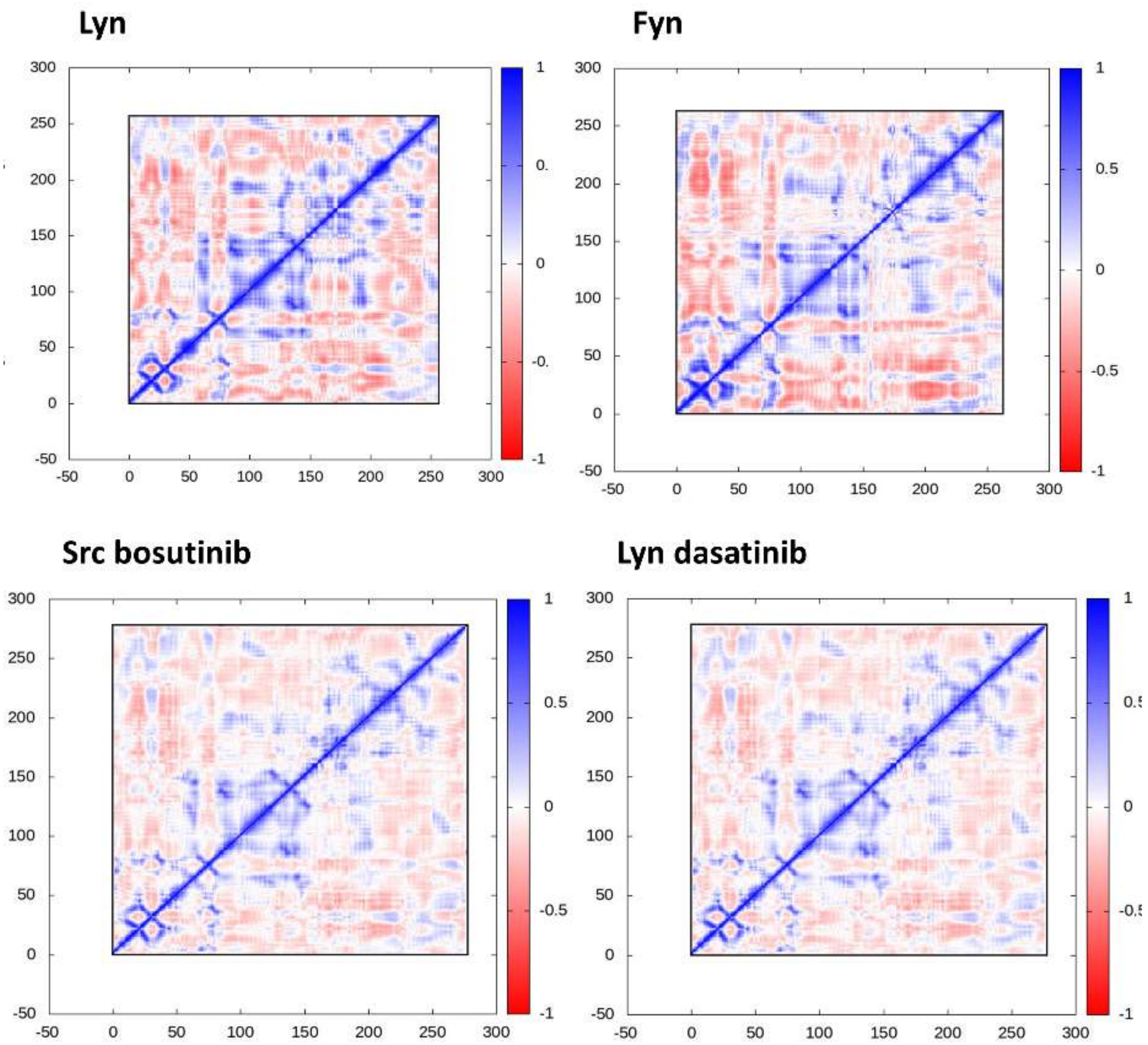

Figure S5. Correlation analysis for Lyn, Fyn, Src-Bosutinib and Lyn-dasatinib Src kinase structures.

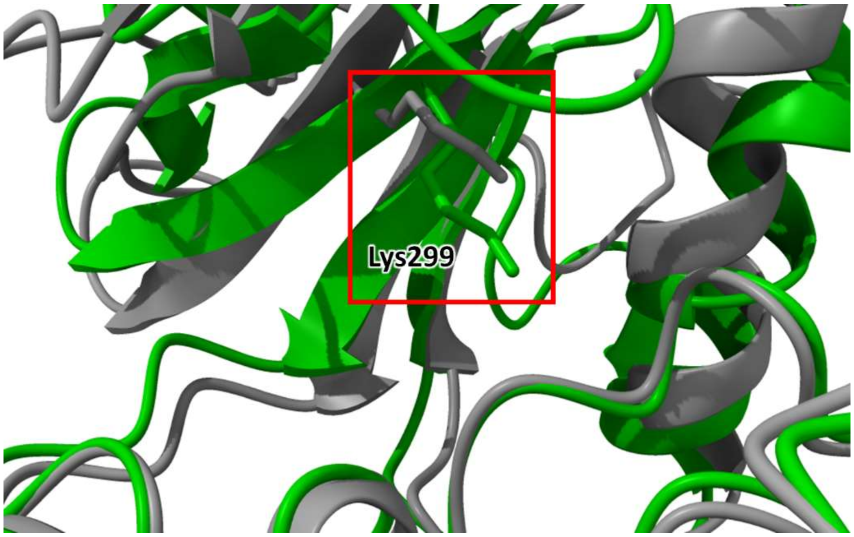

Figure S6. Comparison of the positioning of the important lysine residue next to the Fyn binding site between the crystal structure (gray) and snapshot from the simulation trajectory (green). The residue resides in a lower position, blocking water molecules and affecting water pharmacophore generation.

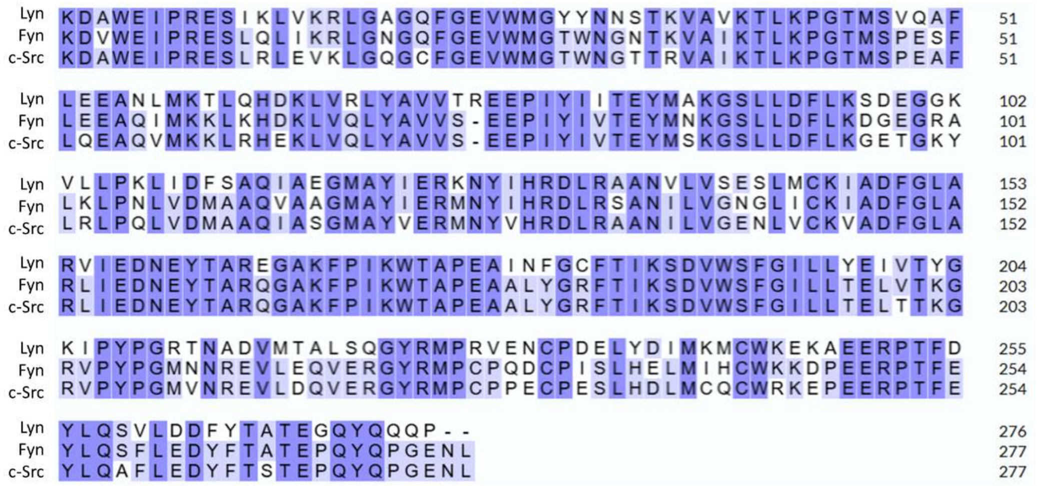

Figure S7. Multiple sequence alignment for the catalytic domain of Lyn, Fyn and c-Src kinases.

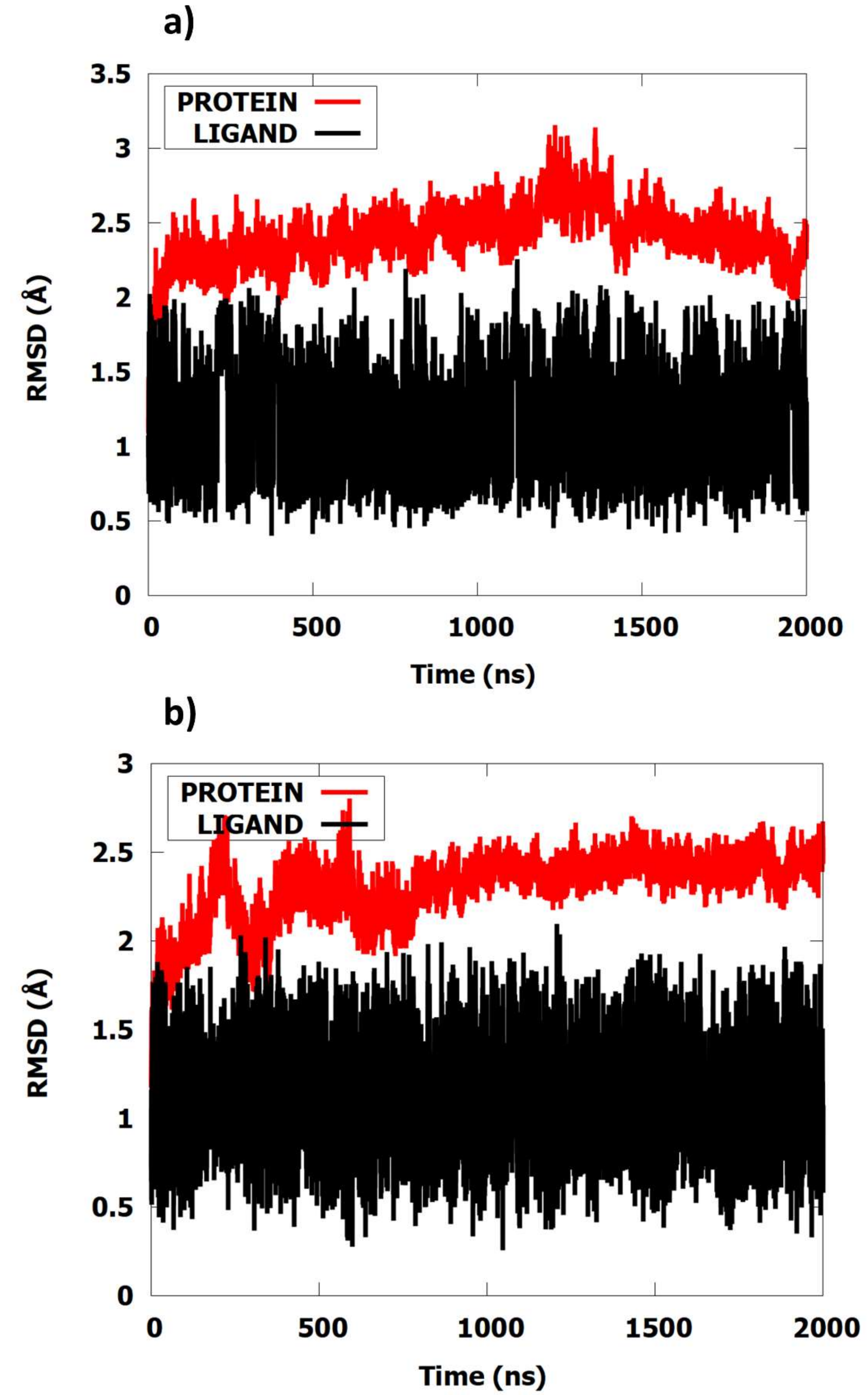

Figure S8. RMSD values (protein in black and ligand in red) for a) the bosutinib-Src and b) dasatinib-Lyn systems during 2000 ns long MD simulations runs.

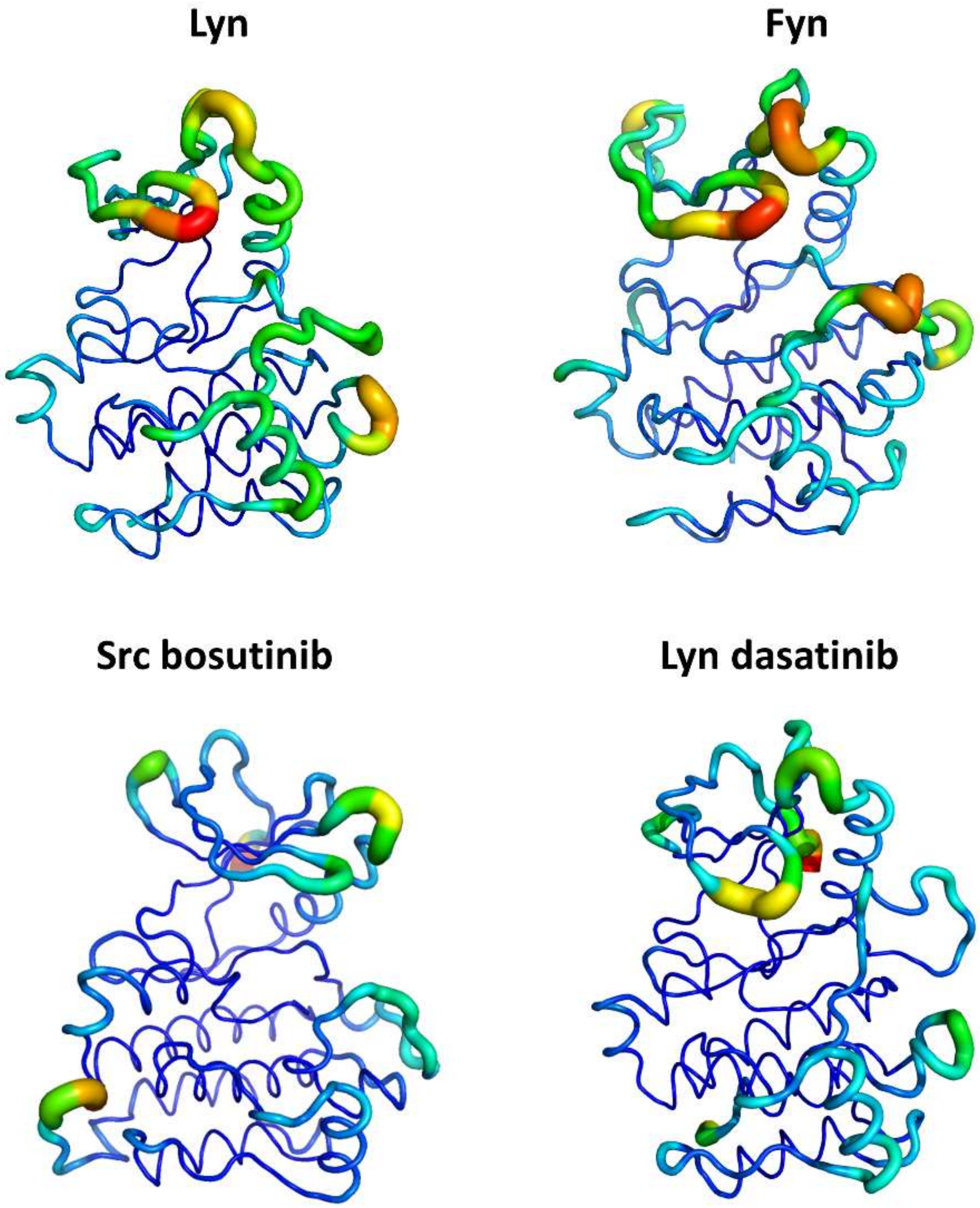

Figure S9. B-factor flexibility representations, projected onto the same starting structure of the Lck, Lyn, Fyn, Lck inactive, Src-Bosutinib and Lyn-dasatinib kinase structures.

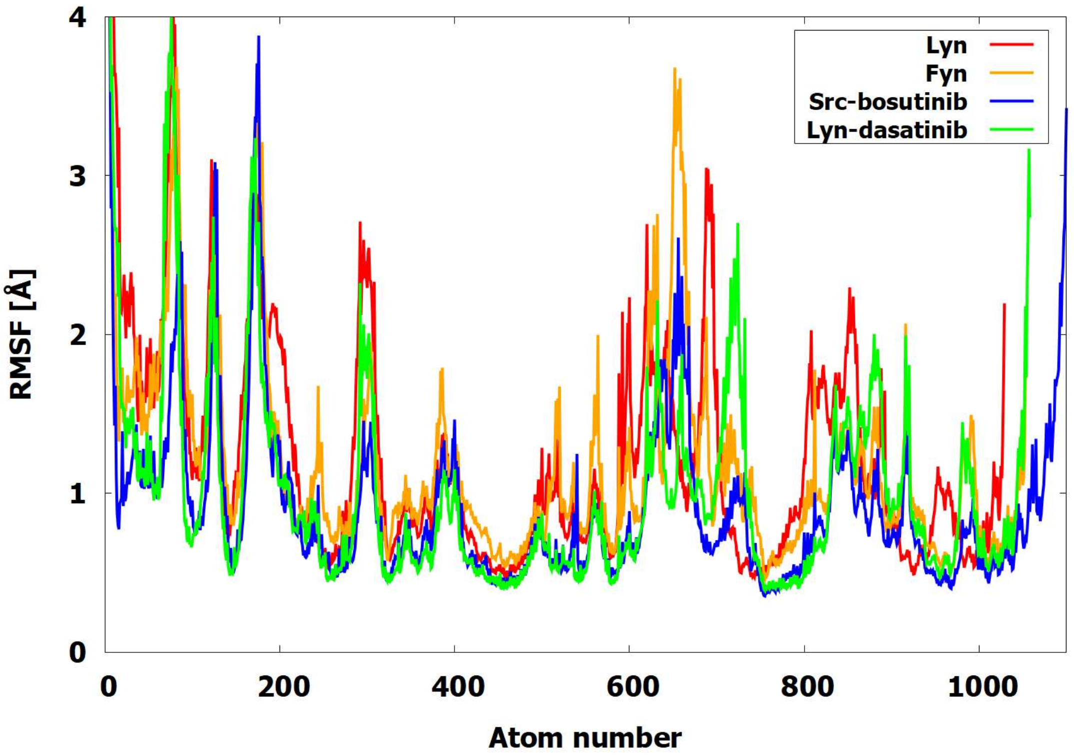

Figure S10. RMSF comparison between the Lyn (red), Fyn (orange), Src-Bosutinib (blue) and Lyn-dasatinib (green) simulated models.

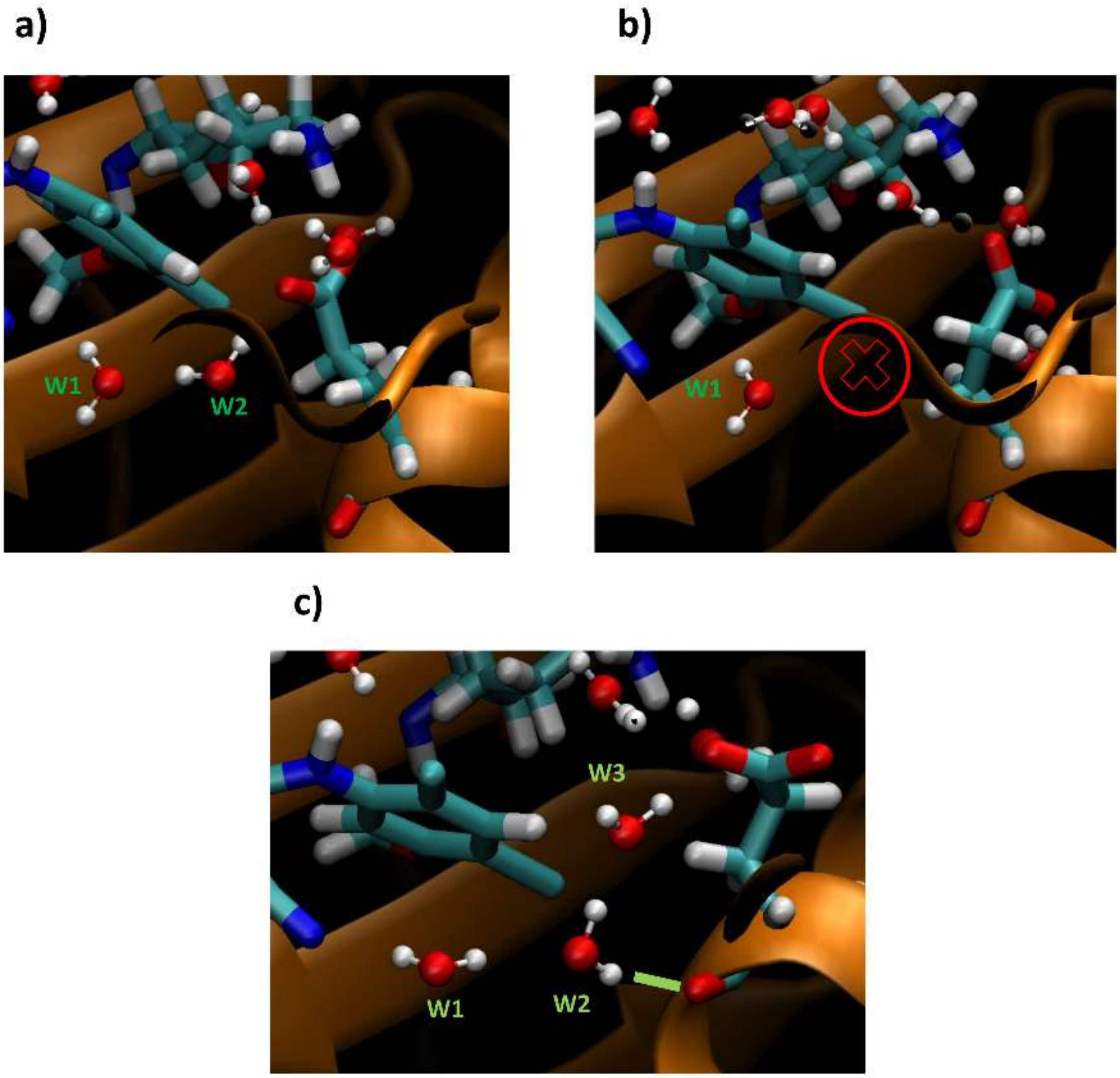

Figure S11. Representative snapshots of the Src-bosutinib binding site which showcases the water molecule positioning around the ligand structure. a) Water bridge consisting of two water molecules between the bosutinib nitrile group and Glu314 of the kinase, b) missing water bridge with only one molecule present and c) water network which includes an additional water molecule forming an H-bond with Glu314.

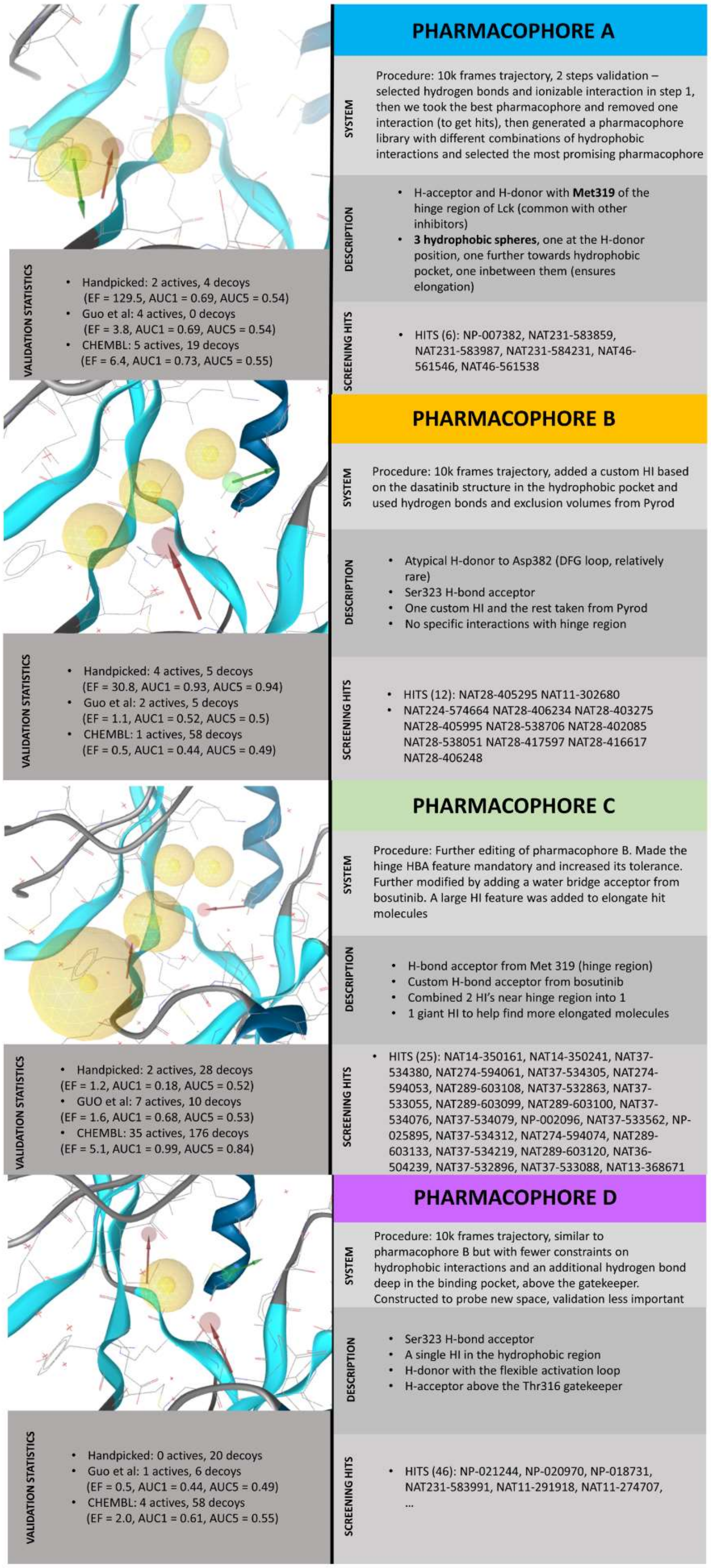

Figure S12. Details regarding the three selected pharmacophores for the active kinase form, including validation statistics.

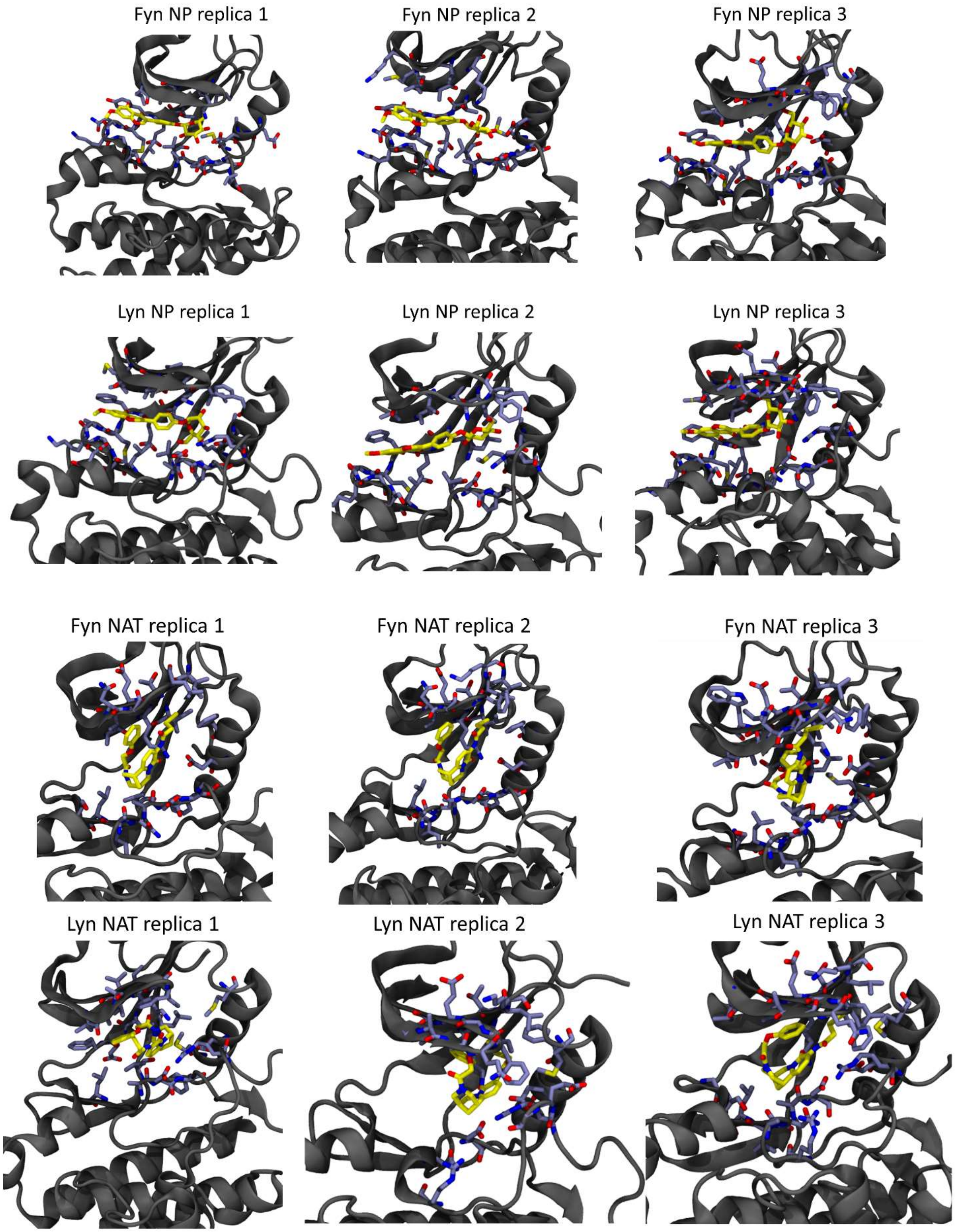

Figure S13. Representative structures of compounds **1** and **2** (yellow) in all replica simulations.

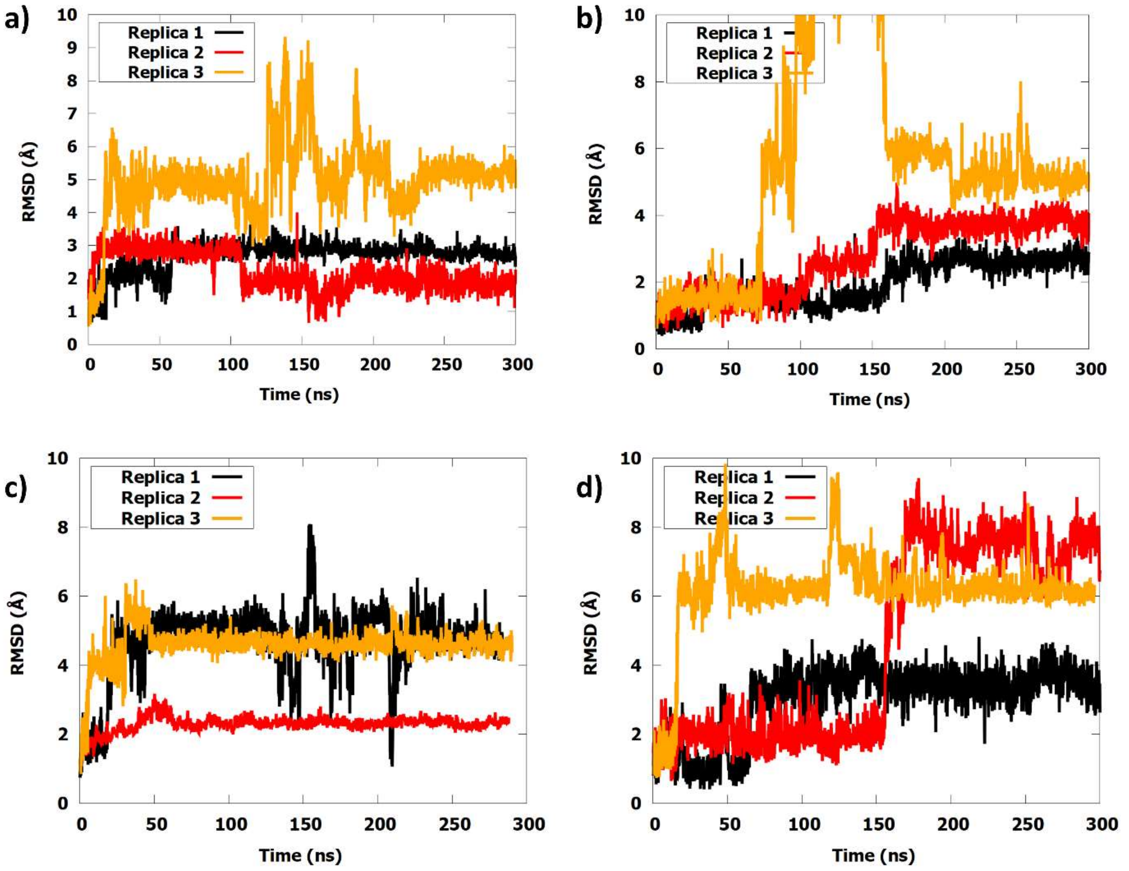

Figure S14. RMSD values of compounds **1** and **2** inside the binding pockets of Fyn and Lyn. a) Fyn with compound **1**, b) Lyn with compound **1**, c) Fyn with compound **1** and d) Lyn with compound **2**. Replicas 1, 2 and 3 are presented with black, red and orange plot lines, respectively.

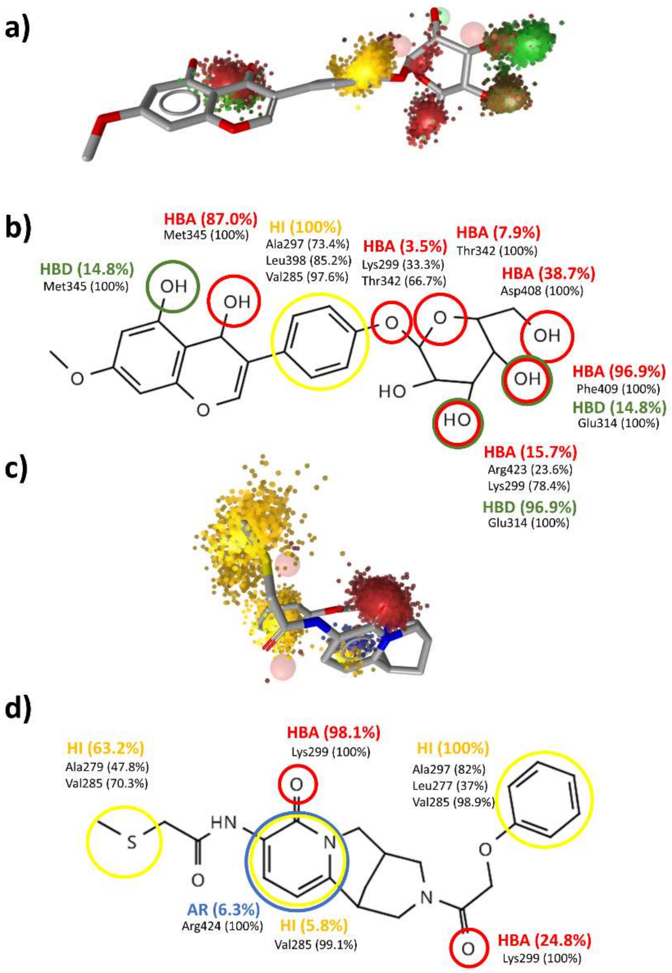

Figure S15. Dynophore models of compounds **1** and **2** for the lowest energy Lyn replicas. a) Dynophore model of compound **1**. b) 2D representation of the compound **1** dynophore model with the interaction pattern constructed from the percentage occurrence of each pharmacophore feature. c) Dynophore model of compound **2** of Lyn. d) 2D representation of the compound **2** dynophore model with the interaction pattern constructed from the percentage occurrence of each pharmacophore feature.

Table S1. A chemically diverse library of small organic molecules from the NATx and MEGx. Analyticon compound libraries, selected for in vitro testing as prospective Fyn and Lyn kinase inhibitors with the pharmacophore they were derived from on the left. Activity values from inhibition assays are displayed on the right. Additionally, we determined the IC_{50} values for the most promising compound (NP-021244).

PH	Library name	Compound	Fyn Activity [%]	Lyn Activity [%]
A	NP-025895	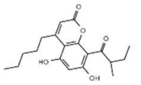	133	81
A	NAT231-583987	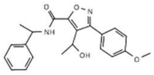	129	101
A	NAT46-561538	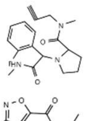	122	83
A	NAT231-584231	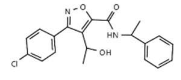	90	87
B	NAT28-416617	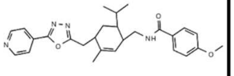	112	83
B	NAT28-405295	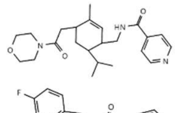	125	81
B	NAT224-574664	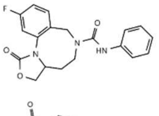	113	89
B	NAT11-302680	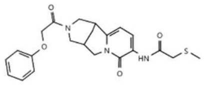	**50**	74
C	NAT289-603108	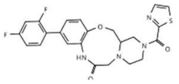	109	75
C	NAT289-603133	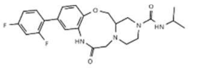	135	92
C	NAT37-535362	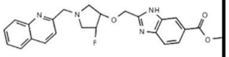	75	93
C	NAT37-534380	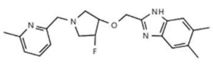	98	117
C	NAT13-368671	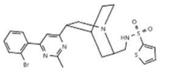	93	81
C	NAT14-350241	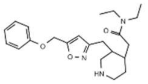	94	97
C	NAT274-594061	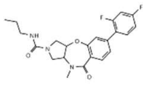	88	84
D	NP-021244	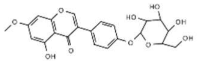	**42**<br>**IC_{50} = 8.66 μM**	**5**<br>**IC_{50} = 4,39 μM**

Table S2. MMGBSA energy values, calculated using every 10th frame from the second half of each replica simulation. The averaged values for each system are shown on the right.

SYSTEM	ENERGY [kcal/mol]	AVERAGES [kcal/mol]
Fyn NP 1	-39.4 ± 4.9	
Fyn NP 2	-37.8 ± 5.7	**-36.5**
Fyn NP 3	-32.2 ± 3.3	
Lyn NP 1	-48.6 ± 3.9	
Lyn NP 2	-40.4 ± 4.1	**-39.4**
Lyn NP 3	-29.2 ± 5.5	
Fyn NAT 1	-24.9 ± 3.8	
Fyn NAT 2	-26.8 ± 2.9	**-29.7**
Fyn NAT 3	-37.3 ± 3.1	
Lyn NAT 1	-34.2 ± 3.3	
Lyn NAT 2	-30.2 ± 3.3	**-30.8**
Lyn NAT 3	-28.1 ± 2.9	

Analaytical data for active hit compounds:

Compound **1**

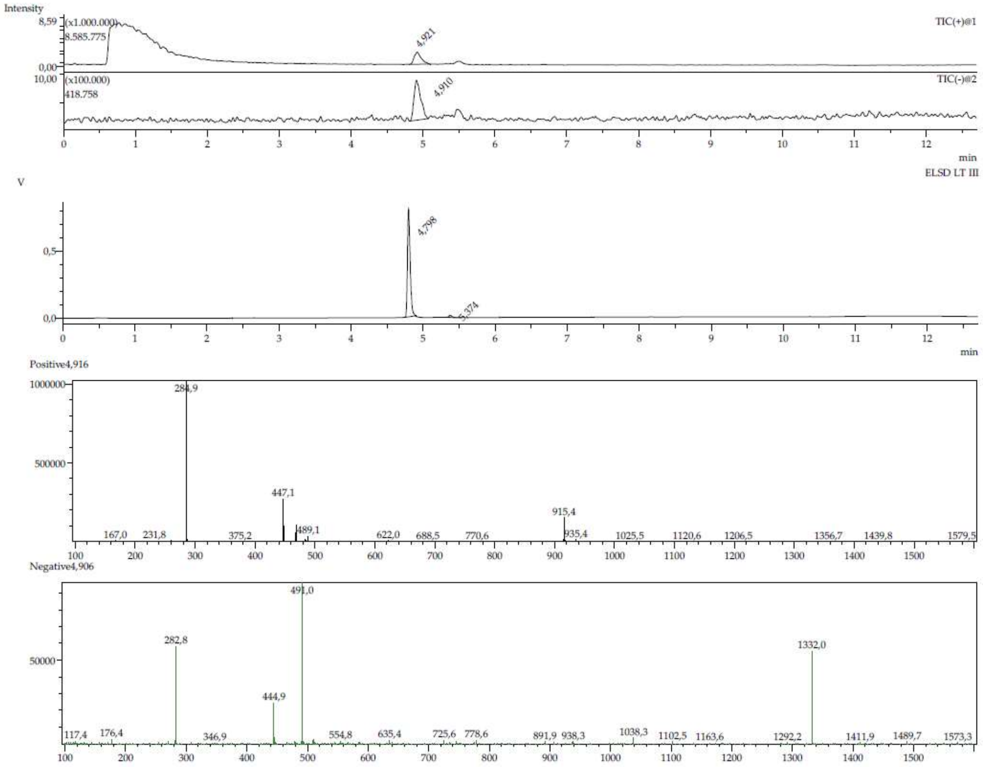

Peak Table H-2359-K-06_NF1_MEGx_Store_MTP-1832_220209
ELSD

Peak#	Ret. Time	Area	Area%
1	4,798	2089894	98,040
2	5,374	41777	1,960
Total		2131671	100,000

Compound **2**

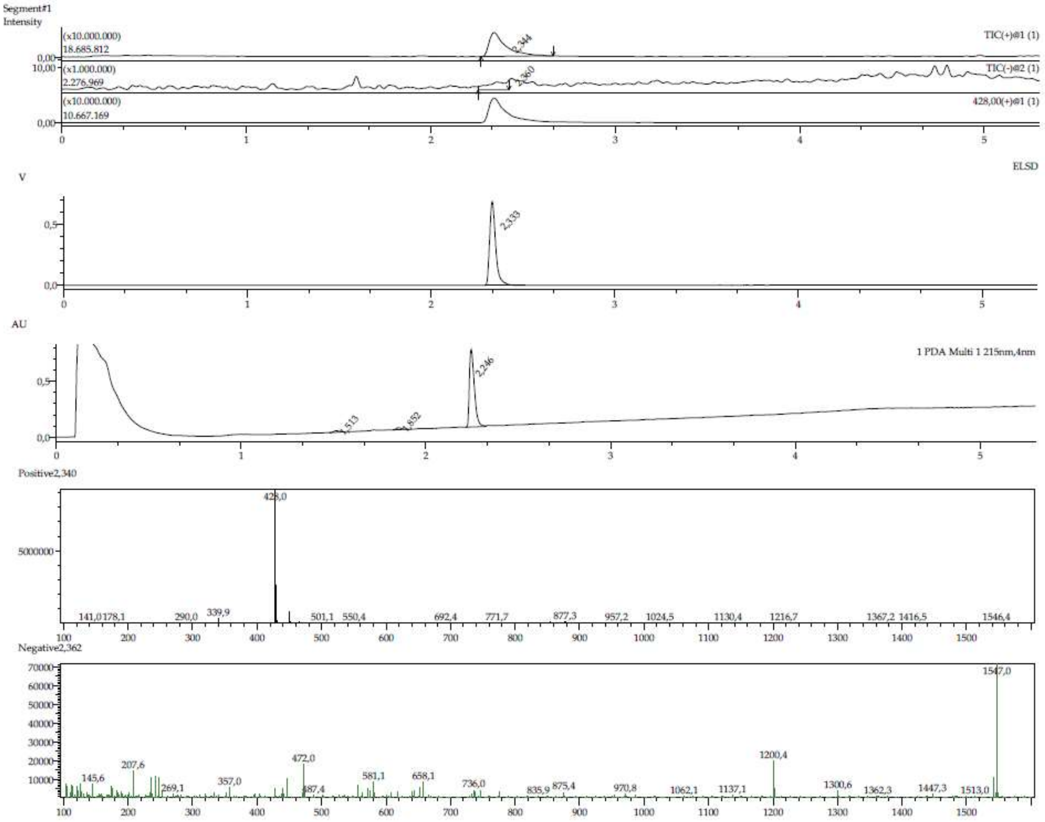

Peak Table NATx_Store_MTP-1373_C04_180611
AD2

Peak#	Ret. Time	Area	Area%
1	2,333	1553854	100,000
Total		1553854	100,000

PDA Ch1 215nm

Peak#	Ret. Time	Area	Area%
1	1,513	25052	1,840
2	1,852	35342	2,595
3	2,246	1301488	95,565
Total		1361882	100,000
